# Honey Bee Infecting Lake Sinai Viruses

**DOI:** 10.3390/v7062772

**Published:** 2015-06-23

**Authors:** Katie F. Daughenbaugh, Madison Martin, Laura M. Brutscher, Ian Cavigli, Emma Garcia, Matt Lavin, Michelle L. Flenniken

**Affiliations:** 1 Department of Plant Sciences and Plant Pathology, Montana State University, Bozeman, MT 59717, USA; E-Mails: kdaughenbaugh@gmail.com (K.F.D.); mpm.91@live.com (M.M.); laura.brutscher@msu.montana.edu (L.M.B.); icavigli@gmail.com (I.C.); celticgarcia@gmail.com (E.G.); mlavin@montana.edu (M.L.); 2 Institute on Ecosystems, Montana State University, Bozeman, MT 59717, USA; 3 Department of Microbiology and Immunology, Montana State University, Bozeman, MT 59717, USA

**Keywords:** honey bee, honey bee virus, *Apis mellifera*, Lake Sinai virus

## Abstract

Honey bees are critical pollinators of important agricultural crops. Recently, high annual losses of honey bee colonies have prompted further investigation of honey bee infecting viruses. To better characterize the recently discovered and very prevalent Lake Sinai virus (LSV) group, we sequenced currently circulating LSVs, performed phylogenetic analysis, and obtained images of LSV2. Sequence analysis resulted in extension of the LSV1 and LSV2 genomes, the first detection of LSV4 in the US, and the discovery of LSV6 and LSV7. We detected LSV1 and LSV2 in the *Varroa destructor* mite, and determined that a large proportion of LSV2 is found in the honey bee gut, suggesting that vector-mediated, food-associated, and/or fecal-oral routes may be important for LSV dissemination. Pathogen-specific quantitative PCR data, obtained from samples collected during a small-scale monitoring project, revealed that LSV2, LSV1, Black queen cell virus (BQCV), and *Nosema ceranae* were more abundant in weak colonies than strong colonies within this sample cohort. Together, these results enhance our current understanding of LSVs and illustrate the importance of future studies aimed at investigating the role of LSVs and other pathogens on honey bee health at both the individual and colony levels.

## 1. Introduction

Throughout the globe, insects pollinate plant species that enhance landscape biodiversity and agricultural crops valued at $153 billion annually [1]. Honey bees (*Apis mellifera*) are the primary pollinators of US crops, valued over $17 billion per year [2]. Therefore, high annual honey bee colony mortality in North America and in some parts of Europe concern beekeepers, growers, scientists, and government officials [3,4,5]. In the US, annual losses of honey bee colonies have averaged ~32% since 2006 [6,7,8,9,10]. Research to date suggests that multiple abiotic and biotic factors affect colony health [9,11,12,13]. Although no single factor is responsible for colony losses or Colony Collapse Disorder (CCD), honey bee samples from CCD-affected colonies had higher pathogen (e.g., viruses and *Nosema*) prevalence and abundance [10,14,15,16]. Longitudinal monitoring of colony health and pathogen prevalence and abundance is critical for determining the role of pathogens in colony losses [11,17,18,19,20,21]. Recent colony monitoring efforts coupled with molecular diagnostic techniques and next generation sequencing resulted in the discovery of additional honey bee infecting viruses, including the Lake Sinai viruses [14,20,22,23,24].

The majority of honey bee infecting viruses are small (+) sense RNA viruses of the *Picornavirales* order [5,22,25,26,27,28], and unclassified RNA viruses including Chronic bee paralysis virus (CBPV) [29] and the Lake Sinai viruses (LSVs) [14,20,23,24,30]. Lake Sinai virus 1 (LSV1) and LSV2 were discovered in honey bee samples obtained from a migratory commercial beekeeping operation with sites near Lake Sinai, South Dakota, US [20]. These viruses were the most abundant pathogens detected in a 10 month honey bee pathogen monitoring study carried out in the US in 2008–2009 [20]. In that sample cohort, LSV2 was the most abundant virus with peak levels in April and January, whereas LSV1 infections peaked in July [20]. While the pathogenicity of LSVs is not well understood, LSV1 and LSV2 loads were higher in Colony Collapse Disorder (CCD) affected colonies, as compared to unaffected colonies [14]. Since the discovery of LSV1 and LSV2, the LSV group has been expanded to include LSV3 [14], LSV-Navarra [23], LSV4, LSV5, and several LSVs discovered in Belgium [24,30]. LSVs have been detected in the US, Spain, Belgium, and Turkey [14,20,23,24,30,31], as well as in multiple bee species [32].

Lake Sinai virus genomes were first identified via high throughput sequencing of libraries generated from RNA isolated from virus-enhanced honey bee lysates [20]. LSVs are likely ssRNA viruses that replicate via a negative-strand RNA intermediate; the replicative form is readily detected by strand-specific PCR [20,24]. The RNA genomes of LSV1 and LSV2 are approximately 5.6 kb in length and share 71% identity at the nucleotide (nt) level [20]. LSV2 and LSVs isolated in Belgium in 2015 contain a variable spacer (19–23 nt) between the capsid protein and the RNA dependent RNA polymerase (RdRp) protein encoding regions, whereas these protein encoding sequences overlap in LSV1 [20,30]. The nucleotide binding pocket (*i.e.*, DxSRFD and SG amino acid motifs), and all eight conserved viral RdRp domains are present in the RdRp encoded by all sequenced LSV genomes [20,30,33]. Phylogenetic analyses based on the RdRp protein sequences of Lake Sinai viruses indicated that they are most similar to Chronic bee paralysis virus (CBPV) and form their own monophyletic clade that is distinct from the *Nodaviridae* family, which includes plant, fish, and insect-infecting viruses [20,22,25,30]. Interestingly, analyses based on virus capsid proteins indicated that LSVs share the highest capsid amino acid sequence identity with Nudaurelia capensis beta-virus (18% identity) and a recently described mosquito infecting virus, Mosinovirus (32%–34% identity) [20,25].

To further characterize this group of viruses we sequenced LSVs currently circulating in Western US honey bee populations, performed phylogenetic analysis, and purified LSV2. Sequence analysis resulted in extension of the LSV1 and LSV2 genomes, extension of the LSV4 RNA dependent RNA polymerase (RdRp) sequence, and identification of LSV6 (GenBank KR021357) and LSV7 (GenBank KR021355). The presence of the replicative intermediate forms of LSV1 and LSV2 in honey bee samples were confirmed by negative-strand specific PCR. To better understand the distribution and transmission of LSVs, we determined the prevalence and abundance of LSVs in honey bee colonies located in Montana and California, examined the distribution of LSV2 in individual bees, and screened mites for LSVs. We determined that LSVs were very common and abundant in bee samples, and detected LSV1 and LSV2 in *Varroa destructor* mites. In honey bees LSV2 levels were highest in gut tissue, readily detected in the thorax and abdomen, and present at low levels in the head. Lastly, to begin to investigate a potential role of LSVs and other pathogens on honey bee colony health, we monitored colony health (using colony size as proxy for health) and the prevalence and abundance of common honey bee pathogens. LSV2 and LSV1 were the most abundant pathogens evaluated in this sample cohort and the abundance of these and other pathogens (*i.e.*, BQCV and *Nosema ceranae*) was greater in weak or collapsed colonies as compared to healthy or recovered colonies. This result underscores the importance of including LSVs in future longitudinal monitoring studies aimed at determining the role of pathogens on honey bee colony health.

## 2. Results

### 2.1. Lake Sinai Viruses Are Readily Detected in Honey Bee Samples

**Figure 1:**
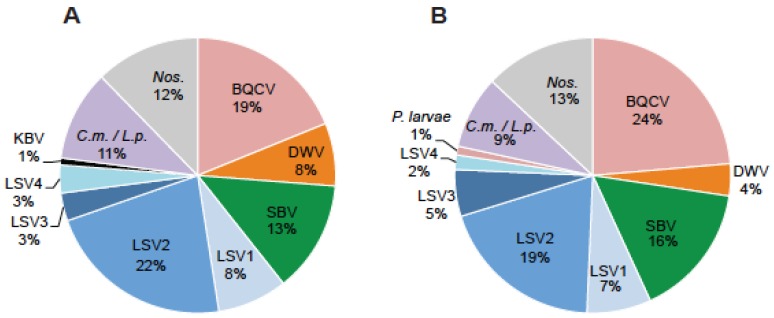
**Distribution of honey bee pathogens detected in weak and strong colonies.** Honey bee samples were obtained from 54 monitor colonies from October 2013 to June 2014. PCR was used to test for 16 honey bee infecting pathogens including: viruses (ABPV, BQCV, CBPV, DWV, IAPV, KBV, SBV, LSV1, LSV2, LSV3, LSV4, and LSV5, microsporidia (*Nosema spp*.; *Nos.*), bacteria (*P. larvae* and *M. plutonius*), and trypanosomatids (*Crithidia mellificae, C.m.*/*Lotmaria passim L.p.*). The pathogen distribution in (**A**) weak (<5 frames; *n =* 41) or (**B**) strong (>9 frames; *n =* 81) honey bee colonies is shown as a percentage of the total number of pathogen-specific PCR tests.

To determine which honey bee pathogens were most common in the Western US, honey bee samples (*n =* 203) were obtained from commercially managed colonies from October 2013 to June 2014 (*n =* 54). Colony strength, using colony population size as a proxy for strength, was measured at each sample collection event. We defined weak colonies as those that had less than five frames covered with bees at the time of sampling (*n =* 41) and strong colonies as those that had nine or more frames covered with bees at the time of sampling (*n =* 81). We tested these samples for 16 honey bee pathogens (LSV1, LSV2, LSV3, LSV4, LSV5, Black queen cell virus (BQCV), Deformed wing virus (DWV), Sacbrood virus (SBV), Chronic bee paralysis virus (CBPV), Israeli acute paralysis virus (IAPV), Kashmir bee virus (KBV), Acute bee paralysis virus (ABPV), *Nosema* spp., *Paenibacillus larvae*, *Melissococcus plutonius*, and trypanosomatids (*i.e.*, *Crithidia mellificae*/*Lotmaria passim* [34,35]) using pathogen-specific PCR [36]. There were 122 positive pathogen-specific PCR tests in weak colonies and 292 positive pathogen-specific PCR tests in strong colonies. We determined that LSVs were common and accounted for 37% of the total positive tests in weak colonies and 34% of positive tests in strong colonies (Figure 1). We confirmed that LSV1 and LSV2 were active infections by performing negative strand specific RT-PCR to detect the replicative intermediate form of the virus genomes (Supplementary Figure S1).

### 2.2. Lake Sinai Virus Genome Sequences Extended

**Figure 2:**
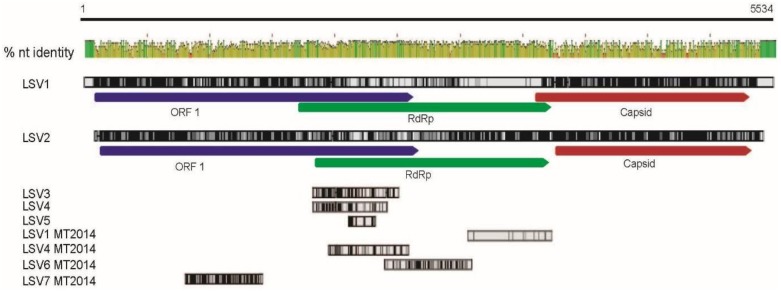
**Lake Sinai viruses—genome comparison and LSV1 and LSV2 genomes extended.** Lake Sinai virus nucleotide alignments using LSV1 (HQ871931) as the reference genome for numbering. The consensus sequence view, high peak height and green color represent maximum nucleotide identity of 100%, low peak height and red color indicate low nucleotide identity. Lake Sinai virus sequences (LSV1, Lake Sinai virus 1 (HQ871931); LSV2, Lake Sinai virus 2 (HQ888865); LSV3, Lake Sinai virus 3 (JQ480620); LSV4, Lake Sinai virus 4 (JX878492); LSV5, Lake Sinai virus 5 (KC880124), and 2014MT sequences: LSV1 (KR021356), LSV4 (KP892556), LSV6 (KR021357), and LSV7 (KR021355). Additional LSV sequences that were included in the Geneious nucleotide alignment using the default cost matrix (65% similarity (5.0/−4.0) [37]), but not depicted in this figure include LSV-Navarra, additional LSV5 sequences, and LSV-genomes from Belgium (Supplementary Table S2).

The majority of the LSV1 and LSV2 virus genomes were sequenced in 2011 (*i.e.*, LSV1, HQ871931, 5508 nt, and LSV2, HQ888865, 5355 nt) (Figure 2). Since the 5′ and 3′ ends of RNA viruses are often critical to virus replication and translation, we sequenced 5′ and 3′ RACE (Rapid Amplification of cDNA Ends) products generated from RNA isolated from LSV-infected bees and semi-purified virus preparations. The 5′end of the LSV2 genome was extended by 54 nt (GenBank KR022002), while no additional LSV1 sequence was obtained. The full-length LSV2 5′ RACE sequence (250 nt) product obtained in this study is 95% identical to the 5′end of LSV2 (HQ888865), including 196 nt of Orf1 (see Supplementary Figure S2). Current evidence, including RT-PCR and sequence information, indicates that LSV genomes are not polyadenylated; therefore to obtain additional 3′ sequence a polyA tail was added *in vitro*. Analysis of LSV1 and LSV2 3′ RACE products resulted in the extension of their genomes by 396 nt and 501 nt, respectively (Table 1, GenBank KR022003, KR022004, Supplementary Figures S3 and S4). We confirmed that these were bona fide LSV1 and LSV2 3′ends that shared >95% identity with existing genomes (Supplementary Figures S3 and S4). The new 3′end sequences of LSV1 and LSV2 share 70% nucleotide identity, similar to the overall percent identity of their entire genomes.

**Table 1:** **New Lake Sinai virus sequences.** The genomes of honey bee infecting viruses, LSV1 (HQ871931, 5508 nt) and LSV2 (5355 nt, HQ888865) were extended by sequencing 5′ and 3′ RACE products generated from RNA isolated from LSV-infected honey bees and semi-purified virus preparations. Sequencing LSVs also resulted additional LSV4 (KP892556) sequence and the discovery of LSV6 (KR021357) and LSV7 (KR021355).

Sequence Name	Accession Number	Read Length	New Seq. Length
LSV1 3′end	KR022003	611 nt	396 nt
LSV2 5′end	KR022002	280 nt	54 nt
LSV2 3′end	KR022004	645 nt	501 nt
LSV1 2014 MT	KR021356	659 nt	n/a
LSV4 2014MT	KP892556	576 nt	129 nt
LSV6 2014MT	KR021357	684 nt	684 nt
LSV7 2015 MT	KR021355	604 nt	604 nt

To characterize the diversity of currently circulating LSVs, we sequenced the RNA-dependent RNA polymerase (RdRp) and Orf1 encoding regions (Table 1, and Supplementary Table S1). We detected LSV1, LSV2, LSV3, and LSV4 in honey bee samples obtained from colonies located in the Western US (*i.e.*, Montana and California), but did not detect LSV5. We sequenced 576 nt of the RdRp encoding region of LSV4 2014MT, isolated from honey bees sampled in Montana (MT) in 2014 (GenBank KP892556). This is the first report of LSV4 in the US, and we extended the previously characterized LSV4 (GenBank JX878492) sequence by 164 nt. The overlapping 460 nucleotides (nt) of LSV4 2014MT and LSV4 are 94.3% identical (Supplementary Figure S5), and 92.8% identical at the amino acid level (Supplementary Table S3). We sequenced 659 nt of the RdRp of a currently circulating LSV1 (LSV1 2014MT, GenBank KR021356). LSV1 2014MT is 97.7% identical at the nucleotide level to LSV1 (HQ871931, Supplementary Table S2) and 98.6% identical at the amino acid level (Supplementary Table S3), and corresponds to LSV1 3010–3669 nts. Our efforts to obtain additional RdRp sequence data also resulted in the discovery of a new LSV sequence (LSV6, GenBank KR021357), which corresponds to the RdRp encoding region of LSV1 (nt 2354–3038). LSV6 is divergent from previously characterized LSVs, sharing only 82.5% nucleotide identity with LSV1 and 75.3% nucleotide identity with LSV2 over this region, corresponding to 92.5% and 82.4% amino acid identity, respectively (Supplementary Table S3). LSV6 shares only 75 nt positions with LSV3 (JQ480620), and these nucleotides are 87.5% identical over that short region. Furthermore, LSV6 shares between 76.5% and 82.6% identity with new LSV genomes discovered in Belgian samples [30] (Figure 2 and Supplementary Table S2). We also discovered LSV7 (GenBank KR021355) based on a 604 nt sequence that corresponds to LSV1 Orf1 coding sequence (*i.e.*, nts 86–1390). LSV7 is only 63.2% identical to LSV1 and 65.3% identical to LSV2. LSV7 is less than 62%–65.1% identical to new LSV genomes obtained from Belgian samples [30] (Figure 2 and Supplementary Table S2). To facilitate simultaneous detection of multiple LSVs we developed an LSV primer set that detects LSV1, LSV2, LSV3, and LSV4 (Supplementary Table S1).

### 2.3 Phylogenetic Analysis

**Figure 3:**
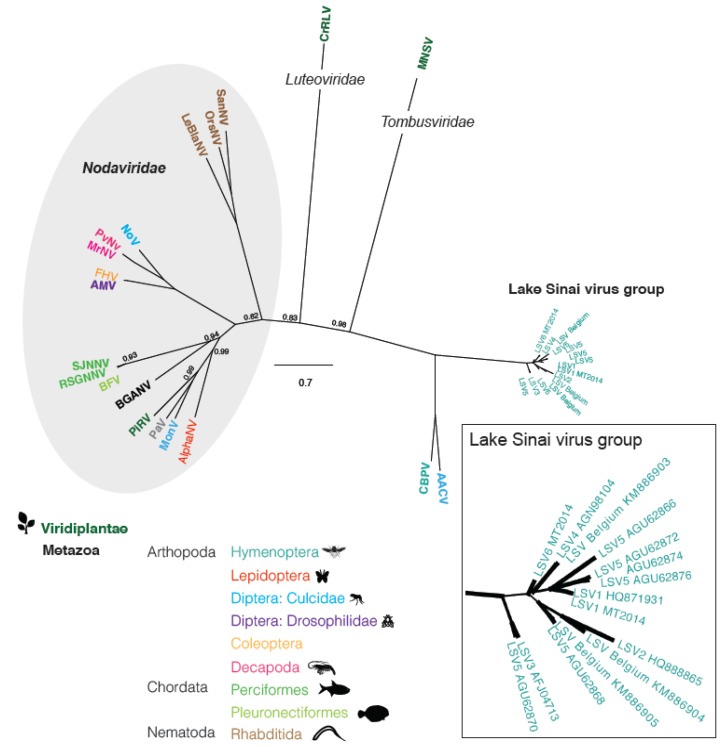
**Lake Sinai virus phylogenetic relationship inferred from RdRp amino acid sequences.** Majority rule Bayesian consensus tree of Lake Sinai viruses derived from Bayesian analysis of an RNA dependent RNA polymerase (RdRp) amino acid alignment implemented in MrBayes v3.1.2 using the WAG amino acid substitution model (Supplementary Figure S6) [39]. Numbers on branches are Bayesian posterior probabilities (0–1). To improve figure clarity only posterior probability values that were less than 1 are shown on the full tree and branch line thickness was used to indicate posterior probabilities (0.5–1) in the LSV inset; the scale bar corresponds to the proportion of amino acid change. GenBank accession numbers (in parentheses) for either the RdRp sequences or the genome sequences from where the RdRp sequence obtained are as follows: **LSV1**, Lake Sinai virus 1 (HQ871931), **LSV1 MT2014** (KR021356), **LSV2** (HQ888865), **LSV3** (AFJ04713), **LSV4** (AGN98104), **LSV Belgium 2015** (KM886905), **LSV Belgium 2015** (KM886903), **LSV Belgium 2015** (KM886904), **LSV6 MT2014** (KR021357), **LSV5 JR** (AGU62868), **LSV5 JR** (AGU62866), **LSV5 JR** (AGU62870), **LSV5 JR** (AGU62872), **LSV5 JR**(AGU62874), **LSV5 JR** (AGU62876), **AACV**, Anopheline-associated C virus RdRp (YP_009011225), **CBPV**, Chronic bee paralysis virus (YP_001911137), **AlphaNV**, Alphanodavirus RdRp (GU976287), **MoNV**, Mosinovirus RdRp (AIO11151), **PaV**, Pariacoto virus RdRp (NC_003691), **PiRV**, Pieris rapae virus RdRp (AY962576), **BGANV**, Bat guano associated nodavirus (HM228873), **BFV**, Barfin flounder nervous necrosis virus RdRp (NC_011063), **SJNNV**, Striped Jack nervous necrosis virus ProtA (NC_003448), **RSGNNV**, Redspotted grouper nervous necrosis virus (AAW32087), **AMV**, Drosophila melanogaster American nodavirus ProtA (GQ342965), **FHV**, Flock house virus RdRp (Q66929), **MrNV**, Macrobrachium rosenbergii nodavirus RdRp (NC_005094), **PvNV**, Penaeus vannamei nodavirus RdRp (NC_014978), **NoV** Nodamura virus RdRp (NC_002690, NP_077730), **LeBNV**, Le Blanc nodavirus (JQ943579), **OrsNV**, Orsay nodavirus RdRp (HM030970), **SanNV**, Santeuil nodavirus RdRp (NC_015069), **MNSV**, Melon necrotic spot virus RdRp (53276), **CrRLV**, and Carrot red leaf virus RdRp (YP_077186).

We examined the relationship between LSVs and other plant and animal infecting viruses by performing phylogenetic analyses based on the virus RNA dependent RNA polymerase (RdRp) amino acid (aa) sequence (Figure 3 and Supplementary Figure S6). The phylogenetic tree derived from this analysis is unrooted, since no suitable outgroup was identified in previous analyses [20,25,30,38]. LSVs are most similar to the honey bee infecting virus Chronic bee paralysis virus (CBPV) and the mosquito-infecting, Anopheline-associated C virus [29,38]. The LSV-containing clade is distinct from the *Nodaviridae* family, which contains insect, plant, decapod crustacean, and nematode infecting viruses. Interestingly, the *Luteoviridae* and *Tombusviridae* virus families, which include numerous viruses that cause plant diseases and are insect-transmitted (e.g., aphids), each form phylogenetically isolated lineages on this RdRp-based phylogenic tree. The 15 LSV RdRp sequences, including both full-length and partial RdRp sequences, did not cluster based on the geographic regions from which they were identified, but did cluster together into a monophyletic clade, which likely warrants designation of a new *Sinaivirus* genus.

### 2.4. Relative Distribution of Lake Sinai Virus 2

**Figure 4:**
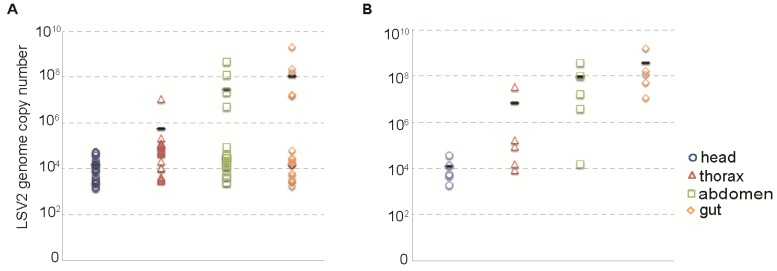
**Relative distribution of LSV2 in honey bees.** (**A**) LSV2-infected adult honey bees (n = 22) were dissected (head, thorax, abdomen, and gut) and the relative abundance of LSV2 was assessed by qPCR. The average copy number per 1 µg RNA of each region (x-axis) is as follows: head—1.56 × 10^4^, thorax—1.96 × 10^6^, abdomen—2.72 × 10^7^, and gut—1.07 × 10^8^ (represented by a black dash); (**B**) Bees with highest LSV2 levels (n = 5) harbored the majority of virus in their gut—4.72 × 10^8^ and abdomen—1.19 × 10^8^ average copy number per 1 µg RNA (black dash), compared to lower copy numbers detected in the thorax—8.42 × 10^6^ and head—1.55 × 10^5^ regions.

The relative distribution of LSV2 in naturally infected bees was assessed by qPCR. Individual bees were dissected into head, thorax, abdomen, and gut fractions. The average LSV2 copy number of each region was determined by qPCR (Figure 4). LSV2 was most abundant in the gut (1.07 × 10^8^ copies/1 µg RNA) and abdomen (2.72 × 10^7^/1 µg RNA) (n = 22). Although reduced, there were readily detectable levels of LSV2 genomes in the heads (1.56 × 10^4^/1 µg RNA) and thoraxes (1.96 × 10^6^/1 µg RNA) of naturally infected honey bees (Figure 4A). These results are most striking in bees with the highest LSV2 infection levels (Figure 4B). In highly infected bees, LSV2 levels in the gut were 30,500× higher than in the head, and 56× higher than the number of copies detected in the thoraxes (*i.e.*, gut—4.72 × 10^8^, abdomen—1.19 × 10^8^, thoraxes 8.42 × 10^6^, heads 1.55 × 10^4^ copies per 1 µg RNA, n = 5) (Figure 4B).

### 2.5. Lake Sinai Virus 2 Purification and Characterization

**Figure 5:**
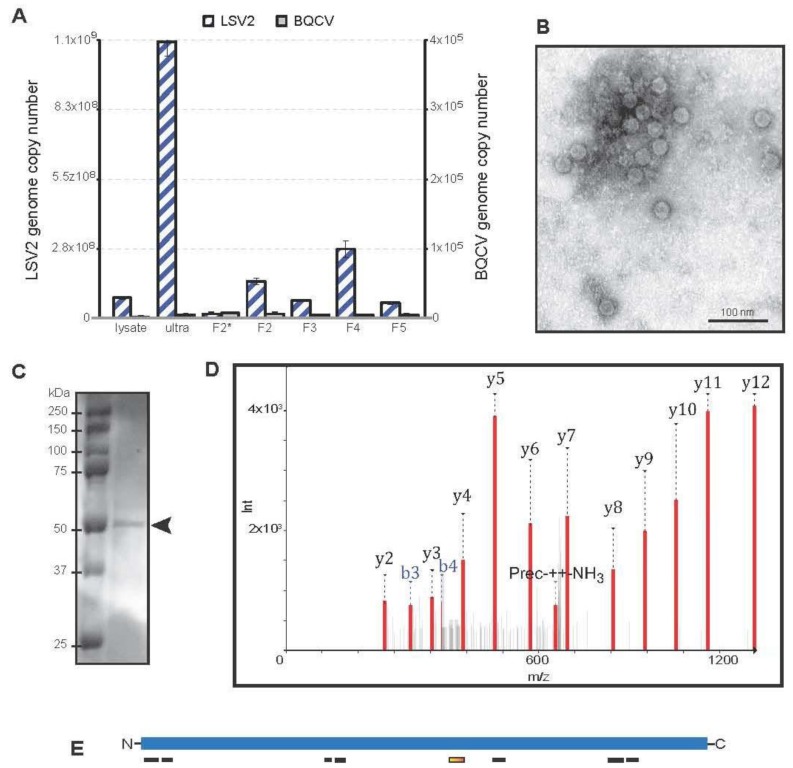
**Characterization of Lake Sinai virus 2 (LSV2).** A standard virus purification protocol was used to isolate honey bee associated viruses from bees primarily infected with LSV2; pathogen specific PCR was used to screen samples for additional pathogens (see Supplementary Figure S7). (**A**) The relative abundance of LSV2 and BQCV in several virus purification subsamples including: the initial honey bee lysate (lysate), virus-pellet after ultracentrifugation (ultra), and several fractions from a CsCl gradient (F2*-fraction 2 unconcentrated, F2—fraction 2 concentrated, F3—fraction 3 concentrated, F4—fraction 4 concentrated, and F5—fraction 5 concentrated), was determined by qPCR. The LSV2 genome copy number per 500 ng RNA for each fraction is as follows: lysate −8.0 × 10^8^, ultra −1.1 × 10^9^, F2* −1.8 × 10^7^, F2 −1.5 × 10^8^, F3 −7.1 × 10^7^, F4 −2.7 × 10^8^, and F5 −6.0 × 10^7^. The BQCV genome copy number per 500 ng RNA for each fraction is as follows: lysate −1.6 × 10^3^, ultra −5.4 × 10^3^, F2* −7.2 × 10^3^, F2 −6.3 × 10^3^, F3 −4.6 × 10^3^, F4 −4.7 × 10^3^, and F5 −5.3 × 10^3^; (**B**) The viruses in fraction 4 (F4), which contained the most LSV2 genome copies (*i.e.*, 2.7 × 10^8^ copies/500ng RNA), were imaged using a TEM (37,000x magnification). The icosahedral virus particles have an average diameter of 27.7 ± 3.1 nm; (**C**) The proteins contained in fraction 4 were analyzed by SDS-PAGE, and a single protein band (arrow) from fraction 4 was visualized by Coomassie staining; (**D**) The putative LSV2 capsid protein (MW 57.3 kDa) band was isolated and analyzed by mass spectrometry. Spectrum and fragment ions from MS peptide1 (NVESSSQTVSSMPR) corresponding to LSV2 capsid protein 286–300 aa (orange rectangle); (**E**) Illustration of peptide matches (rectangles) to the predicted LSV2 capsid protein (blue line). Peptides identified by mass spectrometry covered 18.85% of the LSV2 capsid protein sequence; Supplementary Table S4 includes peptide and LSV2 capsid amino acid sequences.

To better characterize LSV2, we isolated honey bee viruses from bees in which the predominant virus infection was LSV2. Pathogen specific-PCR was used to screen bee samples for 14 pathogens, including LSV2 (Supplementary Figure S7). Honey bees (*n =* 50) from an LSV2-positive colony were homogenized, filtered, and subjected to cesium chloride (CsCl) step-gradient centrifugation to enrich the number of virus particles in specific fractions based on their particle density [40]. In parallel, viruses were directly pelleted from filtered lysate via ultracentrifugation. Since several honey bee viruses, primarily small RNA viruses with icosahedral symmetry, typically co-purify, we tested the ultracentrifuged sample (ultra) and all the fractions from the CsCl gradient for LSV1, LSV2, LSV3, LSV4, Black queen cell virus (BQCV), Deformed wing virus (DWV), Sacbrood virus (SBV), Acute bee paralysis virus (ABPV), Chronic bee paralysis virus (CBPV), Israeli acute paralysis virus (IAPV), and Kashmir bee virus (KBV) by PCR; only LSV2 and BQCV were detected (Supplementary Figure S7) [26]. The relative abundances of LSV2 and BQCV in several virus purification subsamples including the initial honey bee lysate (lysate), virus-pellet after ultracentrifugation (ultra), and several CsCl gradient fractions, were determined by qPCR (genome copy number per 500 ng RNA) (Figure 5). LSV2 genome copy number was highest in the pelleted virus (ultra) and fraction 4 (F4) of the CsCl gradient, with each having 1.09 × 10^9^ and 2.72 × 10^8^ copies, respectively (Figure 5A). Relatively low levels of BQCV were detected (*i.e.*, 5.02 × 10^3^ copies in the ultra sample and 4.73 × 10^3^ copies in F4) (Figure 5A).

In order to visualize LSV2 capsids, the viruses in fraction 4 (F4) were imaged using a transmission electron microscope (TEM) at 37,000× magnification. Fraction 4 contained the most LSV2 genome copies and 5477× more LSV2 genome copies than BQCV. The TEM images indicated that LSV2 capsids are icosahedral with an average diameter of 27.7 ± 3 nm (Figure 5B). To further support that F4 contained primarily LSV2 virions, the proteins contained in that fraction were analyzed by SDS-PAGE, and a single protein band was visualized by Coomassie staining (Figure 5C). The putative LSV2 capsid protein (MW 57.3 kDa) band was isolated and analyzed by nano-HPLC-ESI mass spectrometry. An example spectrum and fragment ions from MS are provided for peptide 1 (NVESSSQTVSSMPR), which corresponds to LSV2 capsid protein 286–300 aa (orange rectangle) (Figure 5D–E). Excluding known contaminants (*i.e.*, human keratin), a total of eight unique peptides identified the LSV2 capsid protein in the staining band (Figure 5C–E, Supplementary Table S4). The eight LSV2 corresponding peptides had a Peptide Shaker score and confidence of 100, and covered 18.85% of the LSV2 capsid protein sequence (GI: 335057591) (Figure 5E, Supplementary Table S4). In addition, one peptide corresponding to *Apis mellifera* Major royal jelly protein 2 (UnitProt/Swiss-Prot 077061) was detected (Supplementary Table S4). This protein has a molecular weight similar to the LSV2 capsid protein and is very abundant in bees; therefore it likely contaminated our honey bee virus preparation. No significant non-contaminant hits were identified in the control region of the gel. Together these data indicate that we were able to augment samples for LSV2. Purification of virus isolates is critical to the development of reagents and infectious clones required to further investigate the interactions between LSV and its honey bee host.

### 2.6. LSV1 and LSV2 Detected in the Varroa Destructor Mite

**Figure 6:**
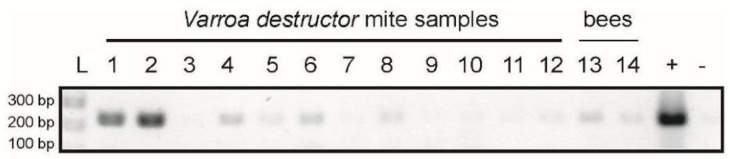
**Lake Sinai virus detection in *Varroa destructor* mites and honey bees.** Lake Sinai virus 2 specific PCR was used to detect LSV2 in mite and bee samples. Lanes 1–12 are individual *Varroa destructor* mite samples, lanes 13 and 14 are honey bee samples (*n =* 5 per sample) from the same colony; molecular weight marker (L), positive control (+), and negative control (−).

To investigate if the ectoparasitic mite *Varroa destructor* harbors LSVs, we screened individual mites for LSV1 and LSV2. We detected LSV1 or LSV2 in 13.2% and 14.7% of mites, respectively (*n =* 68). In one LSV2 positive colony, we detected LSV2 in 58% of the mites tested (*n =* 12) (Figure 6). These data indicate that mites harbor LSV1 and LSV2 and may transmit these viruses, though additional studies are required to determine this and to assess if LSVs replicate in mites.

### 2.7. LSV and Colony Health

**Figure 7:**
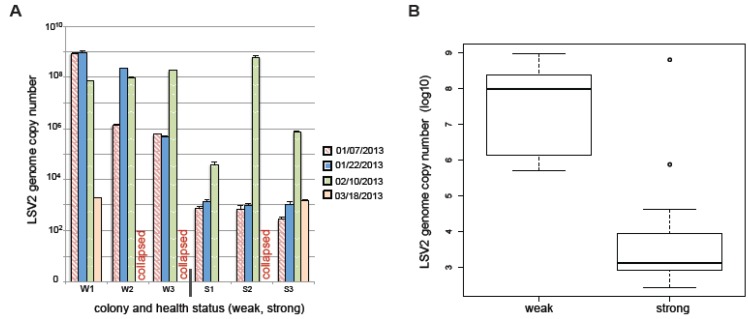
**Lake Sinai virus 2 abundance trends with colony health.** Honey bee colony health and pathogen prevalence and abundance (*n =* 6) were monitored from January–March 2013. Honey bee colonies that were weak (<5 frames, *n =* 3) at the onset of the study are labeled W1, W2, and W3, and colonies that were strong (>9 frames, *n =* 3) at the onset of the study are labeled S1, S2, and S3. (**A**) Quantitative-PCR was used to determine the relative number of LSV2 genomes associated with each sample. Overall weak colonies had higher levels of LSV2 than strong colonies, as did colonies that died during the monitoring study (W2, W3, S2). Recovery of one colony that was weak at the onset of the study (W1) coincided with lower levels of LSV2. (**B**) The mean abundance of LSV2 was greater in weak colonies (log_10_ *=* 7.55) as compared to strong colonies (log_10_ *=* 3.19, Welch Two Sample *t*-test *p =* 8.89 × 10^−5^). In this small sample cohort weak colonies (<5 frames, *n =* 9 sampling events) had greater levels of pathogens relative to strong colonies (>9 frames, *n =* 11 sampling events) (Supplementary Figures S8 and S9).

We monitored six commercially managed colonies located in California from January–March 2013. Pathogen-specific PCR was performed to screen for six pathogens: viruses (BQCV, DWV, LSV1, LSV2), trypanosomatids (*Crithidia mellificae*/*Lotmaria passim*), and the microsporidia *Nosema ceranae* (Supplementary Tables S1 and S5). Colony health was evaluated at each sampling event; colony size was used as a proxy for colony health, and we defined weak colonies as those that had less than five frames covered with bees at the time of sampling (*n =* 9) and strong colonies as those that had nine or more frames covered with bees at the time of sampling (*n =* 11) [41,42]. We detected BQCV, DWV, LSV1, LSV2, and *Nosema ceranae* using pathogen-specific PCR, and performed qPCR analysis for LSV2, LSV1, BQCV, and *Nosema ceranae,* since the majority of samples tested positive for those pathogens (Figure 7 and Supplementary Figure S8). LSV2 and LSV1 were the most abundant pathogens in this sample cohort (Figure 7, Supplementary Figures S8 and S9, and Supplementary Table S5). Overall, the results from this small sample cohort indicate that the abundances of LSV2 (Figure 7), LSV1, BQCV, and *Nosema ceranae* were greater in weak colonies as compared to strong colonies (Supplementary Figures S8 and S9); there was no statistical difference between the abundance of trypanosomatids (*Crithidia mellificae*/*Lotmaria passim*) in weak and strong colonies (Supplementary Figure S9). One colony that was weak at the onset of the study (*i.e.*, W1) but had recovered by the end of the study had a decrease in LSV1 and LSV2 loads while one colony that was strong at the onset of the study but had weakened and was lost at the end of the three-month sampling period (*i.e.*, S2) had greater LSV2, LSV1, and *Nosema ceranae* loads at the end of the study. The data presented herein describe a small sample cohort, therefore extrapolation beyond this population is unwarranted. Additional studies are needed to better understand the effects of pathogens, including the recently discovered LSV virus group, on honey bee health and to assess their potential roles in colony loss.

## 3. Discussion

Lake Sinai viruses are a recently identified, globally distributed group of honey bee infecting viruses. In this study, we sought to further characterize LSVs from the molecular level to the colony level. We sequenced currently circulating LSVs in honey bee populations from Montana and California, which resulted in the extension of the LSV1 and 2 genomes. We also detected LSV4, and discovered LSV6 and LSV7. Phylogenetic analysis based on the virus RdRp sequences indicates that LSVs group in a monophyletic clade that is distinct from Chronic bee paralysis virus and other insect and plant infecting viruses of the *Nodaviridae*, *Luteoviridae*, and *Tombusviridae* families [20,25,38,43]. The diversity of LSVs is remarkable; Ravoet *et al.*, recently demonstrated that individual bees can be infected with LSVs representing three different phylogenetic clades [30]. Likewise, we detected six distinct LSVs (*i.e.*, LSV1, LSV2, LSV3, LSV4, LSV6, and LSV7) from samples obtained from commercial beekeeping operations based in either California or Montana. In addition to honey bees, LSVs infect solitary bee species including *O. cornuta* [32]. Further investigation of the LSV host range and transmission mechanisms are needed to better understand the ecology and evolution of this group of viruses.

*Varroa destructor* mites are major vectors for several honey bee viruses, including DWV and IAPV [44,45,46,47,48]. Since the implications of additional virus transmission by mites to honey bees could be detrimental, we investigated if we could specifically detect LSV1 and LSV2 in *Varroa destructor* mites. We detected both viruses in our samples, corroborating results obtained by Ravoet *et al.* [30]. Ravoet *et al.*, did not detect negative strand LSV, suggesting LSVs may not replicate in mites; however additional studies are needed to further confirm these results and to assess the potential role of mites in LSV transmission. As expected, honey bee viruses have different tissue tropisms. To examine the distribution of LSV2, we dissected bees into head, thorax, abdomen, and gut and assessed viral quantity using qPCR. We determined that bees highly infected with LSV2 had the greatest amount of virus in the gut. Since LSVs have been detected in pollen [30], consumption of contaminated pollen and feeding stored pollen (bee bread) to young bees in the colony could result in the transmission of LSV2. The transmission route(s) of LSVs are unknown and require further investigation.

Both LSV1 and LSV2 abundance have been positively correlated with CCD [14]. In a small sample cohort, we compared LSV abundance to differential colony health (weak or strong, using colony size as a proxy for health) in six colonies over three months and determined that LSV2 abundance was the most statistically different in weak *versus* strong colonies. In addition, LSV1, BQCV, and *Nosema ceranae* abundances were also greater in weak colonies, as compared to strong colonies. Results from this pilot study illustrate the importance of examining the relationship between LSV prevalence and abundance in future longitudinal monitoring studies aimed at determining the relative effects of multiple biotic and abiotic factors on colony health.

Despite the high prevalence and global distribution of the Lake Sinai viruses, the pathology of these viruses is unknown. The development of infectious clones of LSVs would facilitate additional studies that aim to understand the effects of LSVs on honey bees at both the individual bee and colony levels. The data presented herein provide two important steps toward obtaining infectious clones, specifically extension of the LSV1 and LSV2 genomes and isolation of LSV2 from infected honey bees. LSV2 isolation enabled TEM imaging of this virus, which appears to have a capsid with icosahedral symmetry (~27 nm diameter). Characterization of the capsid protein will facilitate the development of antibodies that may prove useful for virus purification and rapid detection. By further describing the molecular characteristics of the LSVs and their interactions with the honey bee host, we may gain additional understanding of these viruses and their potential role in colony losses.

## 4. Methods

### 4.1. Honey Bee and Mite Samples

Honey bees (*Apis mellifera*) and mites (*Varroa destructor*) were collected from privately owned, commercially managed honey bee colonies in Montana, California, and Indiana, US. Samples were collected into plastic bags or tubes, immediately put on dry ice, and stored at −80 °C until RNA extraction. To determine LSV tissue specificity, honey bees were dissected under sterile conditions into head, thorax, abdomen, and whole gut (from honey stomach to hindgut), and RNA was extracted as below.

### 4.2. RNA Isolation

Mites (1 mite/tube) and bees (5 bees/tube) were homogenized in 200–400 uL sterile H_2_O with two sterile borosilicate glass beads (3 mm) by bead beating for 1.5 min in 2 mL tubes, or with a TissueLyzer (Qiagen, Valencia, CA, USA) twice for 2 min at 30 Hz. Bee samples were centrifuged for 15 min at 12,000*×* *g* at 4 °C to pellet debris, and supernatants were transferred to fresh 1.5 mL tubes containing an equal volume of Trizol reagent (Life Technologies, Grand Island, NY, USA). Trizol was added directly to mite homogenate. RNA was extracted according to the manufacturer’s instructions and was suspended in sterile water.

### 4.3. Rapid Amplification of cDNA Ends (RACE)

RACE was performed for LSV1 and LSV2 using the SMARTer RACE cDNA Amplification kit (Clontech, Mountain View, CA, USA) according to the manufacturer’s instructions. cDNA for RACE was prepared as directed using RNA from a natively infected bee sample (LSV1) or from RNA extracted from the LSV2 virus prep (described below). 5′ RACE ready cDNA was prepared for LSV1 and LSV2 using primers included in the kit, and PCR and nested PCR was performed using LSV-1 5′RACE R-273 and LSV-1 5′RACE nest R-428 for LSV1, and LSV-2 5′RACE R-232 and LSV-2 5′RACE nest R-155 for LSV2 (Supplementary Table S1). For 3′ RACE, total RNA was polyadenylated using PolyA Polymerase (Takara, Mountain View, CA, USA) according to the manufacturer’s instructions. PolyA+ RNA was phenol-chloroform extracted and precipitated with ethanol, and 3′RACE ready cDNA was prepared using primers included in the kit. PCR and nested PCR was performed using LSV-1 3′RACE F-5224 and LSV-1 3′RACE nest F-5293 for LSV1, and LSV-2 3′RACE F-5160 and LSV-2 3′RACE nest F-5219 for LSV2. All PCR reactions were performed using the Advantage 2 Polymerase mix (Clontech) according to the manufacturer’s instructions, and were amplified with 25 cycles of the following, depending on the primer set: 94 °C 30 s, 55–68 °C 30 s, 72 °C 3 min. Resulting RACE products were gel purified using the Qiaex II kit (Qiagen) and were cloned into pCR2.1-TOPO (Life Technologies) and cultures were grown at 30 or 37 °C, as some bacteria harboring LSV clones grew best at 30 °C, presumably due to toxicity of insert sequence. Plasmid DNA was purified with the PureYield Plasmid Miniprep System (Promega, Madison, WI, USA) and clones were sequenced with M13F and M13R primers using traditional sequencing methods.

### 4.4. Reverse Transcription/cDNA Synthesis

cDNA synthesis reactions were performed by incubating 250–2000 ng total RNA, M-MLV reverse-transcriptase (Promega) and 500 ng random hexamer primers (IDT, Coralville, IA, USA) for 1 h at 37 °C, according to the manufacturer’s instructions. cDNA was diluted 1:1 with sterile water prior to PCR or qPCR analysis.

### 4.5. Negative Strand-Specific RT-PCR

LSV1 and LSV2 positive samples were analyzed for the presence of negative-strand RNA using strand-specific RT-PCR [20]. RNA from select samples was extracted with Trizol as above, then purified using the RNeasy mini kit (Qiagen), including on-column DNase treatment (Qiagen). cDNA synthesis reactions were performed with SuperScript III (Life Technologies) according to the manufacturer’s instructions using negative strand-specific LSV1 and 2 primers (LSV1-F-1433-TAGS and LSV2-F-1433-TAGS, respectively) tagged with an additional 21 nt of sequence at their 5′ end. The tag sequence (5′GGCCGTCATGGTGGCGAATAA3′) shares no homology with LSV nor to the honey bee genome (Supplementary Table S1). Briefly, 500 ng RNA, hexamer primers, and dNTPs (0.5 mM each) were combined with 10 mL of 1× First-Strand Buffer containing SuperScript III (Life Technologies) (200 U), DTT (5 mM), and RNaseOUT (40 U). Reverse transcription reactions were incubated for 50 min at 50 °C followed by inactivation of the reaction at 85 °C for 5 min. Unincorporated primers present in the RT reactions were digested with 2 units exonuclease I (Life Technologies) per reaction at 37 °C for 30 min, followed by heat inactivation at 85 °C for 5 min. Samples were phenol-chloroform extracted and ethanol precipitated, and PCR was performed using 2 mL of this cDNA template in 25 mcDNA template in 25 mL reactions containing 10 pmol each of a tag-specific forward primer (TAGS) and an LSV1 and LSV2 universal reverse primer (LSV1&2U-Rev-1744) using the following cycling conditions: 95 °C for 5 min; 95 °C for 30 s, 57 °C for 30 s, 72 °C for 30 s, 35 cycles; final elongation 72 °C for 4 min, hold at 4 °C. For negative controls, PCR was performed using template incubated in the absence of RT enzyme during the reverse transcription reaction, and with the reverse primer only. Positive controls included PCR with qPCR primers (qLSV1-F-2569 and qLSV1-R-2743, and qLSV2-F-1722 and qLSV2-R-1947) for detection of LSV1 and LSV2, respectively. Self-priming was tested by performing reverse transcription reactions in the absence of exogenous primers, followed by PCR with qPCR primers as described above. PCR products were analyzed by 1.5% agarose gel electrophoresis and were stained with SYBR safe (Life Technologies).

### 4.6. Polymerase Chain Reaction (PCR)

PCR was performed according to standard methods [49]. In brief, 2 μL cDNA template was combined with 10 pmol of each forward and reverse primer, and amplified with ChoiceTaq polymerase (Denville, South Plainfield, NJ, USA) according to the manufacturer’s instructions using the following cycling conditions: 95 °C for 5 min; 95 °C for 30 s, 57 °C for 30 s, 72 °C for 30 s, 35 cycles, followed by final elongation at 72 °C for 4 min. Select products were Sanger sequenced directly or from TOPO clones generated via ligation of gel-purified products into cloning vectors, as described above, prior to sequencing.

To obtain additional sequence of the LSV4 RdRp, random hexamer-primed cDNA samples were PCR amplified using the Advantage 2 Polymerase mix (Clontech) using primers specific for LSV4 (LSV4-F-1896) and a primer specific for both LSV1 and LSV2 (qLSVU-R-2477), according to the manufacturer’s instructions. Cycling conditions were 94 °C for 30 s; 94 °C 30 s, 57 °C 30 s, 68 °C 30 s, 35 cycles; final elongation at 70 °C for 10 min. PCR product was directly sequenced with the same primers using standard sequencing methods.

### 4.7. Quantitative PCR (qPCR)

qPCR was used to analyze the relative abundance of LSV2 and BQCV in select samples representing distinct stages in virus purification (*i.e.*, initial honey bee lysate (lysate), virus pellet from ultracentrifugation (ultra), and fractions F2*, F2, F3, and F4 from the CsCl gradient). Five hundred ng of RNA from each of these samples was reverse transcribed with M-MLV as described above. All qPCR reactions were performed in triplicate wells with 2 μL of cDNA template. Each well was a 20 μL reaction containing 1× ChoiceTaq Mastermix (Denville), 0.4 μM each forward and reverse primer, 1× SYBR Green (Life Technologies), and 3 mM MgCl2. The plate was run on a CFX Connect Real Time instrument (BioRad, Hercules, CA, USA) with a thermo-profile of a pre-incubation 95 °C for one minute, 40 cycles of 95 °C for 10 s, 58 °C for 20 s, and 72 °C for 15 s, before a melt curve for 65 °C for 5 s to 95 °C before ending. To quantify viral genome copy numbers in the samples, LSV2 or BQCV plasmid standards were used as templates, with titer ranging from 10^9^ to 10^3^ copies to create standard curves for each. The detection limit was 10^3^ copies for both LSV2 (qLSV2-F-1722 and qLSV2-R-1947) and BQCV (qBQCVorf2F_6664 and qBQCVorf2R_6805) primer sets. The host gene *Apis m.* Rpl8 was amplified in triplicate for each sample for comparison, using primers RPL8-Fw1 and RPL8-Rev1. Wells containing no template were run as negative controls. qPCR specificity was verified through melt point analysis and via gel electrophoresis. The linear standard equations for the plasmid standards were Cp *=* −3.7x + 42.1, R^2^ *=* 0.998 for BQCV, and Cp *=* −3.8x + 44.8, R^2^ *=* 0.996 for LSV2.

### 4.8. Honey Bee Pathogen Screening by PCR and qPCR

Honey bee samples (~100 mixed aged bees obtained from the top of frames in the middle of the colony) were collected at each sampling event and five female bees from each sample were used for RNA extraction, cDNA synthesis, pathogen-specific PCR and qPCR [20]. There are varying recommendations on the number of honey bees required to adequately assess the pathogens associated with a single honey bee colony at a particular point in time [19,49,50]. Successful pathogen detection is dependent upon sensitivity of the assay and signal to noise ratio of each sample (*i.e.*, pathogen RNA to bee RNA ratio). The objective for pathogen screening in our study was to identify the most prevalent pathogens associated with honey bees sampled from individual colonies at each sampling event. Based on empirical data, literature values, and practical sample handling considerations, we assayed five bees per colony per sampling event [18,19,20,49,50]. The following equation from Pirk *et al.*, 2013, N *=* ln(1 − D)/ln(1 − P) ((N = sample size, ln = natural logarithm, D = probability of detection, P = proportion of infected bees) predicts that with a sample size of five bees, pathogenic infections affecting 45% or more of the individuals within a colony would be detected with 95% probability [50]. To evaluate the sample size in this study (*i.e.*, five bees/colony per sampling event) and our ability to detect the most common honey bee pathogens from a variable number of bees obtained from a single colony (*i.e.*, 50, 25, 15, 10, 5, and 1) using pathogen-specific PCR (Supplementary Figure S10), we performed three replicate analyses using samples from three different colonies (Supplementary Table S6 and Supplementary Figure S10). Based on the equation above and our results we determined that pathogen screening of five bees per colony produces results that are representative of the majority of the pathogens associated with each colony (Supplementary Figure S10). This result was consistent with previous evaluations of the sample size required to detect the most prevalent bee pathogens associated with samples obtained from one colony sampled multiple times over the course of one study using microarray, PCR, and qPCR [20]. Furthermore, since five bees easily fit into one 2 mL microfuge tube, this sample size avoids potential sample contamination issues that may arise from homogenization of larger samples sizes followed by transfer of homogenized bee tissue (1 gram) into microfuge tubes prior to RNA extraction.

### 4.9. Virus Isolation and Analysis

Viruses were isolated from honey bees obtained from a single colony (Santa Maria, CA, USA, February 2014) using methods similar to those described [20,21,49]. This colony was selected since pathogen-specific PCR screening of five representative bees determined that LSV2 was the predominant pathogen in this colony. Levels of 12 other pathogens including: LSV1, LSV3, LSV4, LSV5, DWV, SBV, *Crithidia mellificae/Lotmaria passim*, *Paenibacillus larvae*, ABPV, IAPV, KBV, *Nosema spp.*, and *Melissococcus plutonius* (listed in Supplementary Table S1) were below the limit of detection. For virus isolation, 50 bees from this colony were ground in liquid nitrogen using a mortar and pestle, transferred to sterile microfuge tubes, and homogenized in PBS pH 7.4 using borosilicate glass beads (3 mm) as describe above. Honey bee cellular debris was removed by centrifugation at 3000*× g* for 15 min at 4 °C, and the supernatant was filtered through a 0.45 μm filter. Half of the virus-containing supernatant (4 mL) was combined with CsCl to a density of 1.1 (207.9 g/L), and then overlaid on top of a CsCl step-gradient of 1.2 (263.3 g/L), 1.25 (322.7 g/L), 1.3 (386.6 g/L), 1.4 (529.6 g/L), and 1.5 (652.7 g/L) in an Ultra-Clear 14 × 89 mm tube (Beckman Coulter, Danvers, MA, USA), as it is likely that most viruses would fall within one of these interfaces [40]. The sample was centrifuged for 2 h at 82,000*× g* (22,000 rpm) using a SW41 rotor at 10 °C (Beckman Coulter Optima L-90K ultracentrifuge). The gradient was fractioned into 1 mL aliquots and each aliquot was concentrated approximately 10-fold using Amicon Ultra-0.5 centrifugal filter units NMWL 100,000 (EMD Millipore) in PBS pH 7.4. Samples were stored at 4 °C or −20 °C. In parallel, the second half of virus-containing supernatant (4 mL) was pelleted by ultracentrifugation in an Ultra-Clear 16 × 76 mm tube (Beckman Coulter) for 3 h at 76,000*× g* (29,000 rpm) using a Type 50 Ti rotor at 10 °C. The pellet was suspended in 100 μL PBS pH 7.4. This sample (ultra) and all gradient samples were tested again for LSV1, LSV2, LSV3, LSV4, BQCV, DWV, SBV, ABPV, CBPV, IAPV, and KBV by PCR, and the relative abundance of LSV2 and BQCV in these samples was assessed by qPCR.

### 4.10. Transmission Electron Microscopy (TEM)

Samples were diluted in water, stained with 2% uranyl acetate and imaged by transmission electron microscopy (TEM) at 15,000× and 37,000× magnification. TEM Leo 912 (Zeiss, Thornwood, NY, USA) equipped with a Proscan CCD camera.

### 4.11. SDS-PAGE

Purified virus was mixed with an equal volume of 2× Laemmli buffer, heated at 95 °C for three minutes, and electrophoresed through a 10% polyacrylamide gel at 150 V for one hour. The gel was stained in Coomassie blue overnight at 4 °C, then destained for 2–4 h at 4 °C. Bands were imaged with a SynGene G: Box gel documentation system.

### 4.12. Phylogenetic Analysis

The Lake Sinai virus containing phylogeny was inferred using Bayesian inference [51,52] as implemented in MrBayes v3.1.2 [39] using an RNA dependent RNA polymerase (RdRp) alignment generated in Geneious R8 [37] using the MAFFT alignment plugin [53] (Figure 3 and Supporting Figure S6). A “mixed-model” approach identified the Whelan and Goldman (WAG) amino acid substitution model with fixed amino-acid frequencies and gamma-shaped rate variation with a proportion of invariable sites, as most explanatory of our data. Metropolis-coupled Markov Chain Monte Carlo (MCMC) permutation of parameters were initiated with a random tree and involved two runs each with four chains set at default temperatures [54]. Markov chains were run for 5,000,000 generations and sampled every 1000 generations. MrBayes identified this sampling rate and a 25% burn-in as sufficient because of the non-autocorrelation of adjacently sampled trees and the complete convergence of the two separate MCMC runs at likelihood stationarity. Trees sampled from post burn-in generations were summarized in a majority rule consensus tree that included posterior probabilities as branch support estimates. The Bayesian majority-rule consensus was then visualized and partially edited using FigTree v1.4.0 (Rambaut, 2012) and Geneious [37]. No suitable outgroup was identified, therefore we opted to present an unrooted tree, consistent with other phylogenetic analysis of this virus group [20,25,30].

GenBank accession numbers for either the RdRp sequences or the genome sequences from where the RdRp sequence obtained are as follows: LSV1, Lake Sinai virus 1 (HQ871931), LSV1 MT2014 (KR021356), LSV2 (HQ888865), LSV3 (AFJ04713), LSV4 (AGN98104), LSV Belgium 2015 (KM886905), LSV Belgium 2015 (KM886903), LSV Belgium 2015 (KM886904), LSV6 MT2014 (KR021357), LSV5 JR (AGU62868), LSV5 JR (AGU62866), LSV5 JR (AGU62870), LSV5 JR (AGU62872), LSV5 JR (AGU62874), LSV5 JR (AGU62876), AACV, Anopheline-associated C virus RdRp (YP_009011225), CBPV, Chronic bee paralysis virus (YP_001911137), AlphaNV, Alphanodavirus RdRp (GU976287), MoNV, Mosinovirus RdRp (AIO11151), PaV, Pariacoto virus RdRp (NC_003691), PiRV, Pieris rapae virus RdRp (AY962576), BGANV, Bat guano associated nodavirus (HM228873), BFV, Barfin flounder nervous necrosis virus RdRp (NC_011063), SJNNV, Striped Jack nervous necrosis virus ProtA (NC_003448), RSGNNV, Redspotted grouper nervous necrosis virus (AAW32087), AMV, Drosophila melanogaster American nodavirus ProtA (GQ342965), FHV, Flock house virus RdRp (Q66929), MrNV, Macrobrachium rosenbergii nodavirus RdRp (NC_005094), PvNV, Penaeus vannamei nodavirus RdRp (NC_014978), NoV Nodamura virus RdRp (NC_002690, NP_077730), LeBNV, Le Blanc nodavirus (JQ943579), OrsNV, Orsay nodavirus RdRp (HM030970), SanNV, Santeuil nodavirus RdRp (NC_015069), MNSV, Melon necrotic spot virus RdRp (53276), CrRLV, Carrot red leaf virus RdRp (YP_077186).

### 4.13. Mass Spectrometry of LSV Capsid Protein

The putative LSV2 capsid protein band (53.7 kDA, Figure 5C) was excised from the acrylamide gel, subjected to in-gel digestion, and the peptide products were detected via nano-HPLC-ESI mass spectrometry. In addition, a non-protein staining control region of the polyacrylamide gel in Figure 5 was processed and analyzed in parallel to our band of interest.

In-gel digestion was performed as described in [55] with minor modifications. To maximize sensitivity, reduction and alkylation steps were omitted, which allowed for fewer sample-handling steps. Triethylammonium bicarbonate (pH 8.5) was used in place of ammonium bicarbonate. After gel piece rehydration with trypsin solution, proteolysis was allowed to proceed overnight at 25 °C. Extraction of the final peptides was performed with two washes of 95/5 (*v/v*) water/acetonitrile with 0.1% formic acid, which were combined and dried, then re-solubilized in 30 µL of the same 95/5 solution for HPLC-MS analysis. Injection volumes of 2 µL were processed via Dionex Ultimate 3000 nano UHPLC, with an Acclaim PepMap100 C18 column used for trapping (100 μm × 2 cm) and an Acclaim PepMap RSLC C18 (75 μm × 15 cm, C18 2 μm 100A) for final peptide separation.

Chromatography was as follows: solvent consisted of H_2_O with 0.1% (*v/v*) formic acid for channel “A” and 80/20 acetonitrile/water for channel “B”. Following sample trapping for 5 min at a flow rate of 10 μL/min, the HPLC valve was switched to elution position. From 0.0 to 5.0 min, the elution solvent pump composition was held at 7% B. From 5.0 to 25.0 min, the elution solvent gradient was linearly changed from 7% to 35% B. From 25 to 27 min, the gradient was ramped from 35% to 95% B. From 27 to 32 min, the solvent was held at 95% B, and from 32 to 33 min the solvent was linearly ramped from 95% B to 7% B. From 33 to 35 min, the elution solvent was held at 7% B. During the entire run, the loading pump solvent was held at 10 μL/min of 97.5% water, 2.5% acetonitrile, and 0.1% formic acid. The mass spectrometer used was a Bruker maXis Impact with CaptiveSpray ESI source: resolution is approximately 50,000 and accuracy is 1 ppm. Spectra were collected in positive mode from 100 to 2500 *m/z* at a maximum rate of 2 Hz for both precursor and fragment spectra, and with adaptive acquisition time for highly-abundant ions. The resulting data files were converted to mzML files with Bruker CompassXport.

Peak lists obtained from MS/MS spectra were identified using X!Tandem version X! Tandem Sledgehammer (2013.09.01.1) [56]. The search was conducted using SearchGUI [57]. Protein identification was conducted against a concatenated target/decoy [58] version of a database that included all *Apis* proteins in TrEMBL, all human proteins in SwissProt, and additional proteins including: Lake Sinai Virus 1 (GenBank: AEH26192.1, LSV1 capsid protein GI:335057599, LSV1 orf1 GI:335057597, LSV1 RdRp GI:335057598), Lake Sinai virus 2 (GenBank: HQ888865.1, LSV2 capsid protein GI:335057591, LSV2 orf1 GI:335057590, LSV2 RdRp GI:335057592), as well as from the open reading frames of two other honey bee viruses Black Queen cell virus (BQCV—2 large poly proteins, NC_003784.1, non-structural protein GI:20451022, structural polyprotein GI:20451023), and DWV (one large poly protein, NC_004830.2, GI:71480056), and one abundant honey bee encoded protein vitellogenin (NM_001011578.1; GI:58585104) complement of the UniProtKB [59], 47247 (target) sequences). The decoy sequences were created by reversing the target sequences in SearchGUI.

The identification settings were as follows: Trypsin with a maximum of two missed cleavages; 20.0 ppm as MS1 and 0.05 Da as MS2 tolerances; variable modifications: oxidation of m (+15.994915 Da), carbamidomethyl c (+57.021464 Da), acetylation of protein n-term (+42.010565 Da), pyro-cmc (−17.026549 Da), pyro-glu from n-term e (−18.010565 Da) and pyro-glu from n-term q (−17.026549 Da). Peptides and proteins were inferred from the spectrum identification results using PeptideShaker version 0.37.5 [60]. Peptide Spectrum Matches (PSMs), peptides and proteins were validated at a 1.0% False Discovery Rate (FDR) estimated using the decoy hit distribution. Post-translational modification localizations were scored using the D-score [61] and the phosphoRS score [62] as implemented in the compomics-utilities package [63]. The mass spectrometry data along with the identification results have been deposited to the ProteomeXchange Consortium [64] via the PRIDE partner repository [65].

Excluding known contaminants (e.g., human keratin), a total of eight unique peptides identified LSV2 capsid protein in the staining band (Figure 4). The eight LSV2 corresponding peptides had a PeptideShaker Score and Confidence of 100, and covered 18.85% of the LSV2 capsid protein sequence (GI: 335057591). In addition, one peptide corresponding to *Apis mellifera* Major royal jelly protein 2 (UnitProt/Swiss-Prot 077061) was detected. This protein has a molecular weight similar to the LSV2 capsid protein and is very abundant in bees; therefore it is likely a contaminating protein in our honey bee virus preparation. No significant non-contaminant hits were identified in the control region of the gel.

## Supplementary Materials

Supplementary materials can be accessed at http://www.mdpi.com/1999-4915/7/6/2772/s1.
