# Supplementary material for: Honey Bee Infecting Lake Sinai Viruses
Source: Viruses. 2015 Jun 23;7(6):3285–309. doi: 10.3390/v7062772 (PMC4488739; doi:10.3390/v7062772)
Supplement: Supplementary file 1 [file viruses-07-02772-s001.zip › viruses-07-02772-supplementary/FigS1 LSV negative strand.pdf]

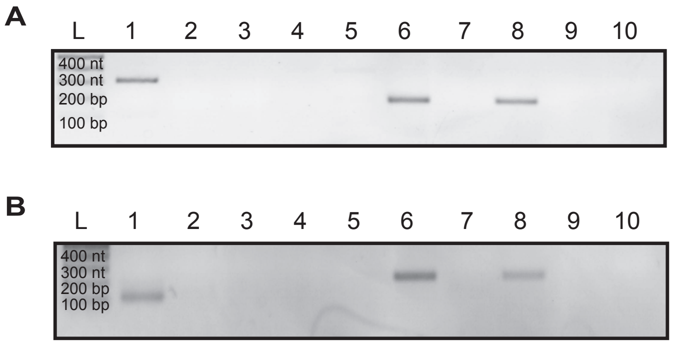

Supplemental Figure S1. LSV1 and LSV2 negative strand detection
RNA isolated from a LSV containing honey bees lysate was reverse-transcribed with the primer listed below, treated with Exonuclease I to remove excess primer, and amplified using the PCR primers listed for each lane:

(A) LSV1 data
(L) molecular weight ladder
(1) RT with tagged-negative strand specific LSV1 primer (LSV1-F-1433-TAGS), PCR with TAGS and LSV1&2U-R-1744 primers
(2) negative control: no RT in the presence of (LSV1-F-1433-TAGS), PCR with TAGS and LSVSV1&2U-R-1744 primers
(3) negative control: RT with tagged-negative strand specific LSV1 primer (LSV1-F-1433-TAGS), PCR with only LSVSV1&2U-R-1744 primer
(4) negative control: RT with random hexamer primer, PCR with TAG and LSVSV1&2U-R-1744 primers
(5) negative control: no RT in the presence of random hexamer primer, PCR with LSV1 qPCR primers (qLSV1-F-2569 and qLSV1-R-2743)
(6) postive control: RT with random hexamer primer, PCR with LSV1 qPCR primers (qLSV1-F-2569 and qLSV1-R-2743)
(7) negative control: RT with random hexamer primer, PCR only reverse LSV qPCR primer (qLSV1-R-2743).
(8) evidence of self priming: RT with no primer, PCR with LSV1 qPCR primers (qLSV1-F-2569 and qLSV1-R-2743)
(9) negative control: no template PCR with TAGS and LSV1&2U-R-1744 primers
(10) negative control: no template PCR with LSV1 qPCR primers (qLSV1-F-2569 and qLSV1-R-2743)

(B) LSV2 data
(L) molecular weight ladder
(1) RT with tagged-negative strand specific LSV2 primer (LSV2-F-1433-TAGS), PCR with TAGS and LSV1&2U-R-1744 primers
(2) negative control: no RT in the presence of (LSV2-F-1433-TAGS), PCR with TAGS and LSV1&2U-R-1744 primers
(3) negative control: RT with tagged-negative strand specific LSV2 primer (LSV2-F-1433-TAGS), PCR with only LSV1&2U-R-1744 primer
(4) negative control: RT with random hexamer primer, PCR with TAGS and LSV1&2U-R-1744 primers
(5) negative control: no RT in the presence of random hexamer primer, PCR with LSV1 qPCR primers (qLSV2-F-1722 and qLSV2-R-1947)
(6) positive control: RT with random hexamer primer, PCR with LSV2 qPCR primers (qLSV2-F-1722 and qLSV2-R-1947)
(7) negative control: RT with random hexamer primer, PCR only reverse LSV2 qPCR primer (qLSV2-R-1947).
(8) evidence of self priming: RT with no primer, PCR with LSV2 qPCR primers (qLSV2-F-1722 and qLSV2-R-1947)
(9) negative control: no template PCR with TAGS and LSV1&2U-R-1744 primers
(10) negative control: no template PCR with LSV2 qPCR primers (qLSV2-F-1722 and qLSV2-R-1947)
