# Supplementary material for: Honey Bee Infecting Lake Sinai Viruses
Source: Viruses. 2015 Jun 23;7(6):3285–309. doi: 10.3390/v7062772 (PMC4488739; doi:10.3390/v7062772)
Supplement: Supplementary file 1 [file viruses-07-02772-s001.zip › viruses-07-02772-supplementary/FigS10 portrait_v4.pdf]

A

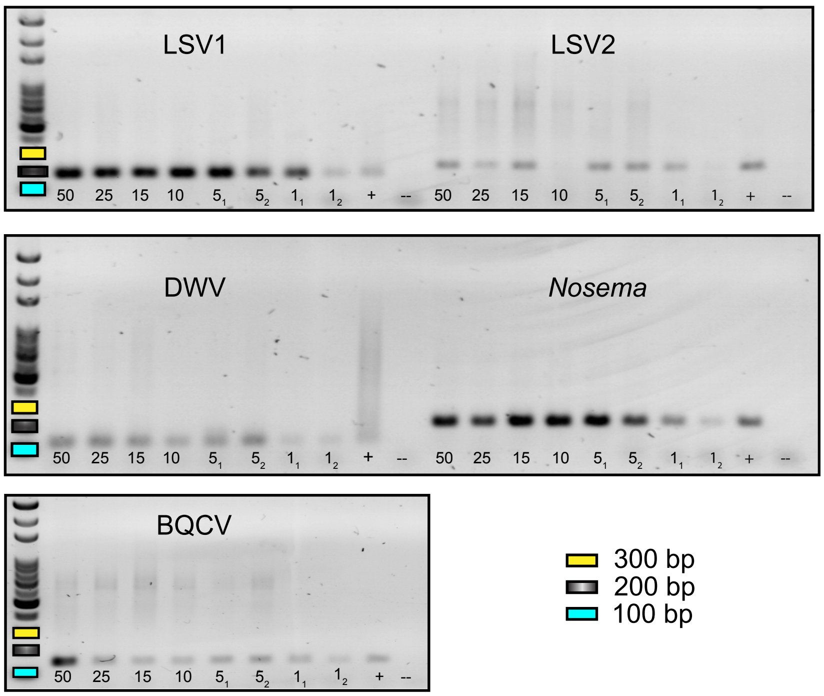


B

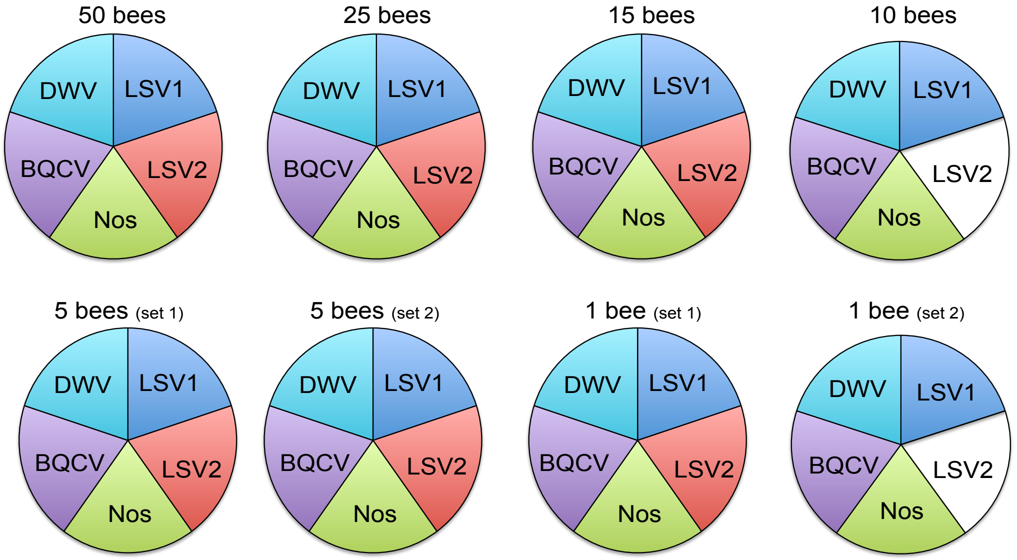


Supplemental Figure S10. **Pathogen detection in RNA samples prepared from variable numbers of honey bees obtained from a single colony.** Different quantities of honey bees (50, 25, 15, 10, 5, or 1) were homogenized (i.e., samples of ≥10 frozen bees were homogenized in sealed plastic bags using a marble rolling pin, then thoroughly mixed by shaking contents, prior to transferring 1 gram of material per sample to a microfuge tube (2 ml) for RNA extraction; samples of ≤5 bees were homogenized in microfuge tubes (2 ml) using 3mm glass beads (see Methods section for additional details)). Following RNA extraction and reverse transcription of a normalized amount of RNA (2000 ng) from each sample, pathogen-specific PCR was performed to identify the pathogens present in each sample. Samples from this representative colony tested positive for BQCV, DWV, LSV1, LSV2, and *Nosema ceranae*.

A. Images of LSV1, LSV2, DWV, *Nosema ceranae*, and BQCV PCR products after agarose gel electrophoresis; sample sizes (i.e., 50, 25, 15, 10, 5(set1), 5(set2), 1(set1), and 1(set2)) are noted in each lane.

B. Graphical representation of pathogens detected in each sample. Colored regions of each pie-chart indicate that the pathogen was detected, whereas white areas indicate that the pathogen was not detected in that sample. The results from this representative experiment of 3 replicates (see Supplemental Table S6), indicate that a sample size of 5 adequately represents the majority of pathogens associated with a particular colony.
