# Supplementary material for: Honey Bee Infecting Lake Sinai Viruses
Source: Viruses. 2015 Jun 23;7(6):3285–309. doi: 10.3390/v7062772 (PMC4488739; doi:10.3390/v7062772)

Supplemental Figure S4. Lake Sinai virus 2 additional 3’end sequence obtained by sequencing 3’ RACE (Rapid Amplification of cDNA ends) products; GenBank KR022004.

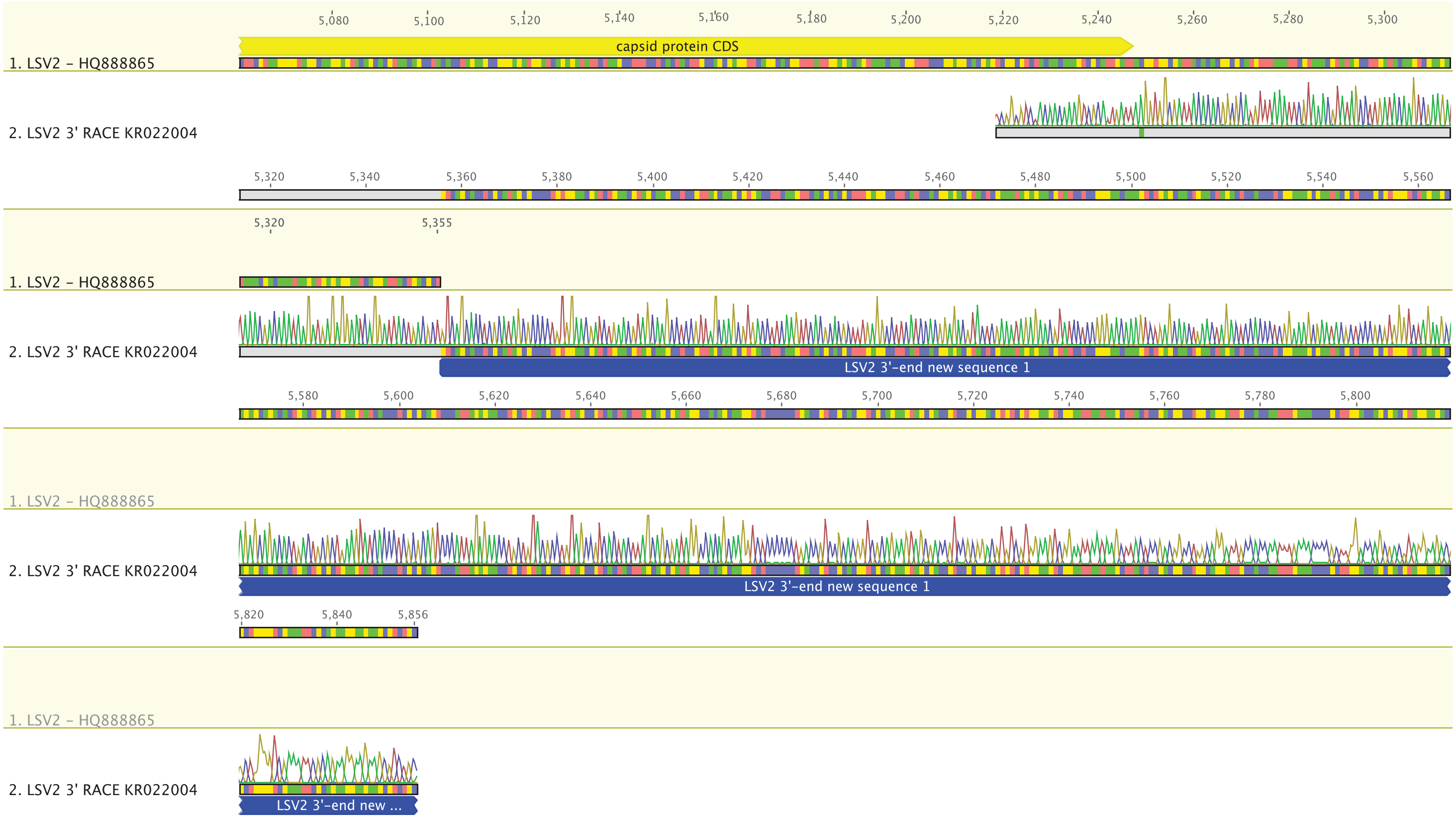

Supplement: Supplementary file 1 [file viruses-07-02772-s001.zip › viruses-07-02772-supplementary/FigS4 LSV2_3end.pdf]
