# Supplementary material for: Honey Bee Infecting Lake Sinai Viruses
Source: Viruses. 2015 Jun 23;7(6):3285–309. doi: 10.3390/v7062772 (PMC4488739; doi:10.3390/v7062772)

Supplemental Figure S5. Lake Sinai virus 4 MT2014 (KR021356), LSV4 (JX878492) and LSV1 (HQ871931) nucleotide sequences were aligned using the Geneious R8 alignment tool.

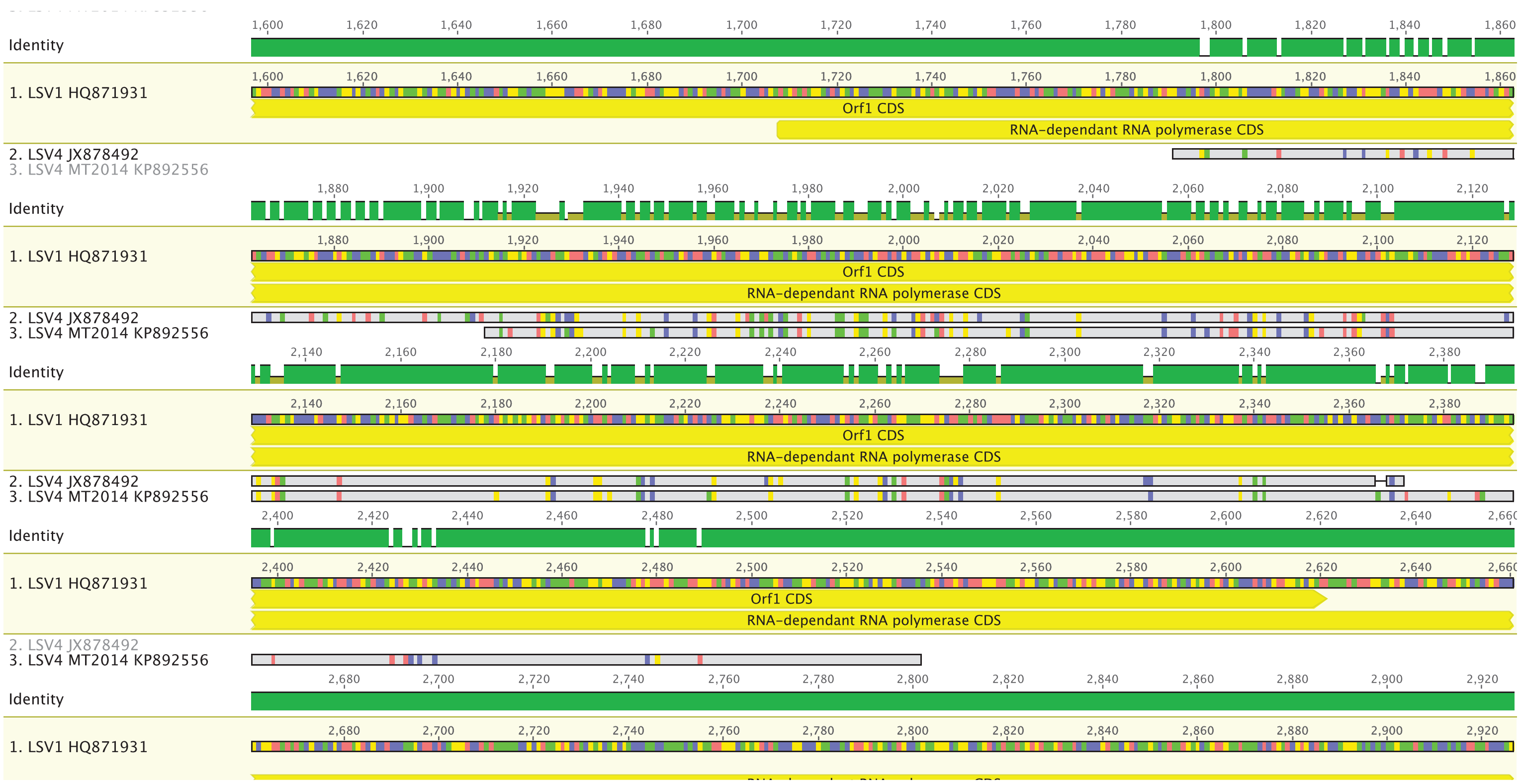

Supplement: Supplementary file 1 [file viruses-07-02772-s001.zip › viruses-07-02772-supplementary/FigS5 LSV4.pdf]
