# Supplementary material for: Honey Bee Infecting Lake Sinai Viruses
Source: Viruses. 2015 Jun 23;7(6):3285–309. doi: 10.3390/v7062772 (PMC4488739; doi:10.3390/v7062772)
Supplement: Supplementary file 1 [file viruses-07-02772-s001.zip › viruses-07-02772-supplementary/FigS6 LSV 1-5 RdRp aa alignment.pdf]

Supplemental Figure S6. Virus RNA-dependent RNA polymerase (RdRp) protein alignment

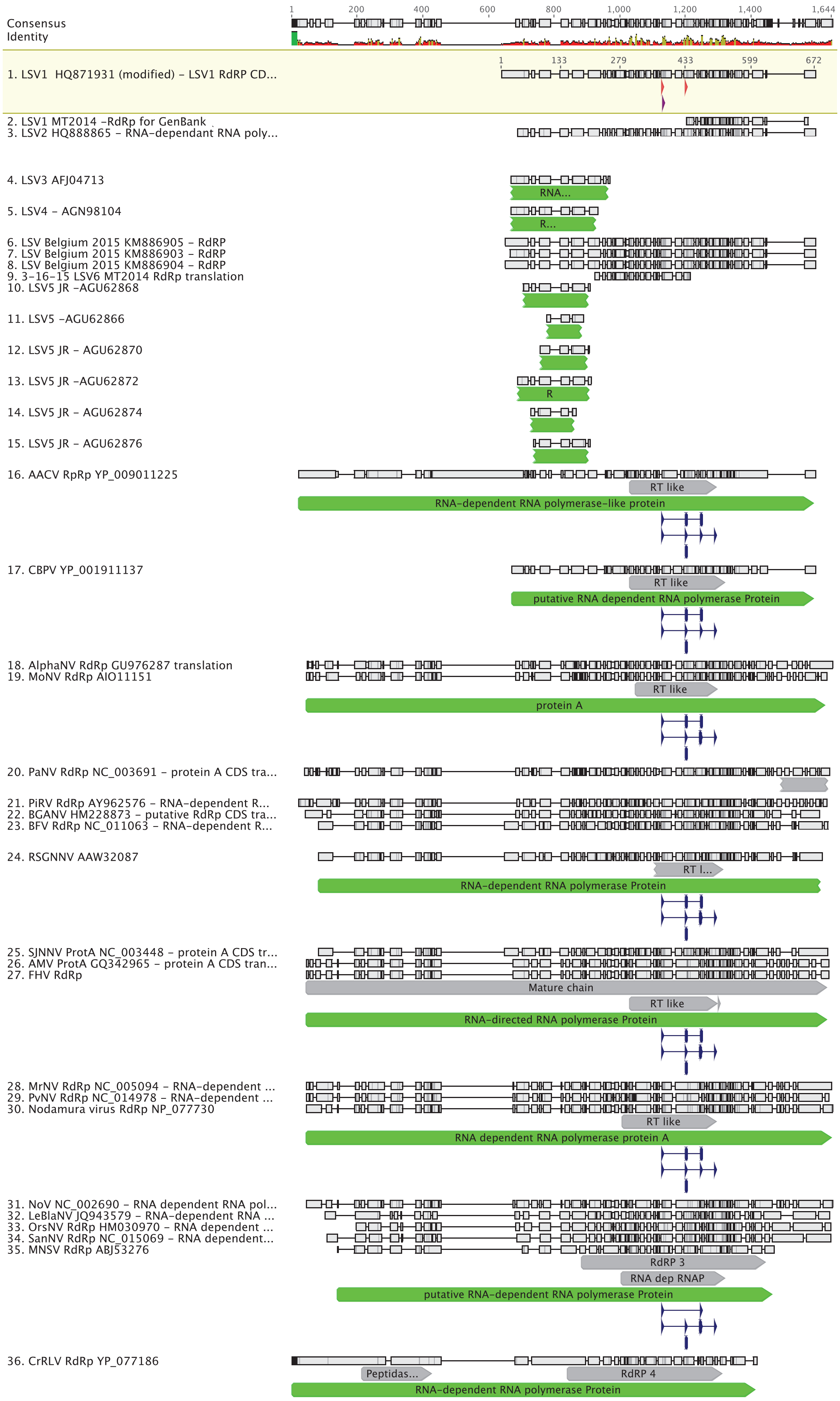

Supplemental Figure S6. Virus RNA dependent RNA polymerase (RdRp) amino acid sequences were aligned generated in Geneious R8 using the MAFFT alignment plugin. GenBank accession numbers are listed in figure and in the methods section.
