# Supplementary material for: Honey Bee Infecting Lake Sinai Viruses
Source: Viruses. 2015 Jun 23;7(6):3285–309. doi: 10.3390/v7062772 (PMC4488739; doi:10.3390/v7062772)
Supplement: Supplementary file 1 [file viruses-07-02772-s001.zip › viruses-07-02772-supplementary/FigS7 LSV virus prep Pathogen Diagnostic SupFigure_v6.pdf]

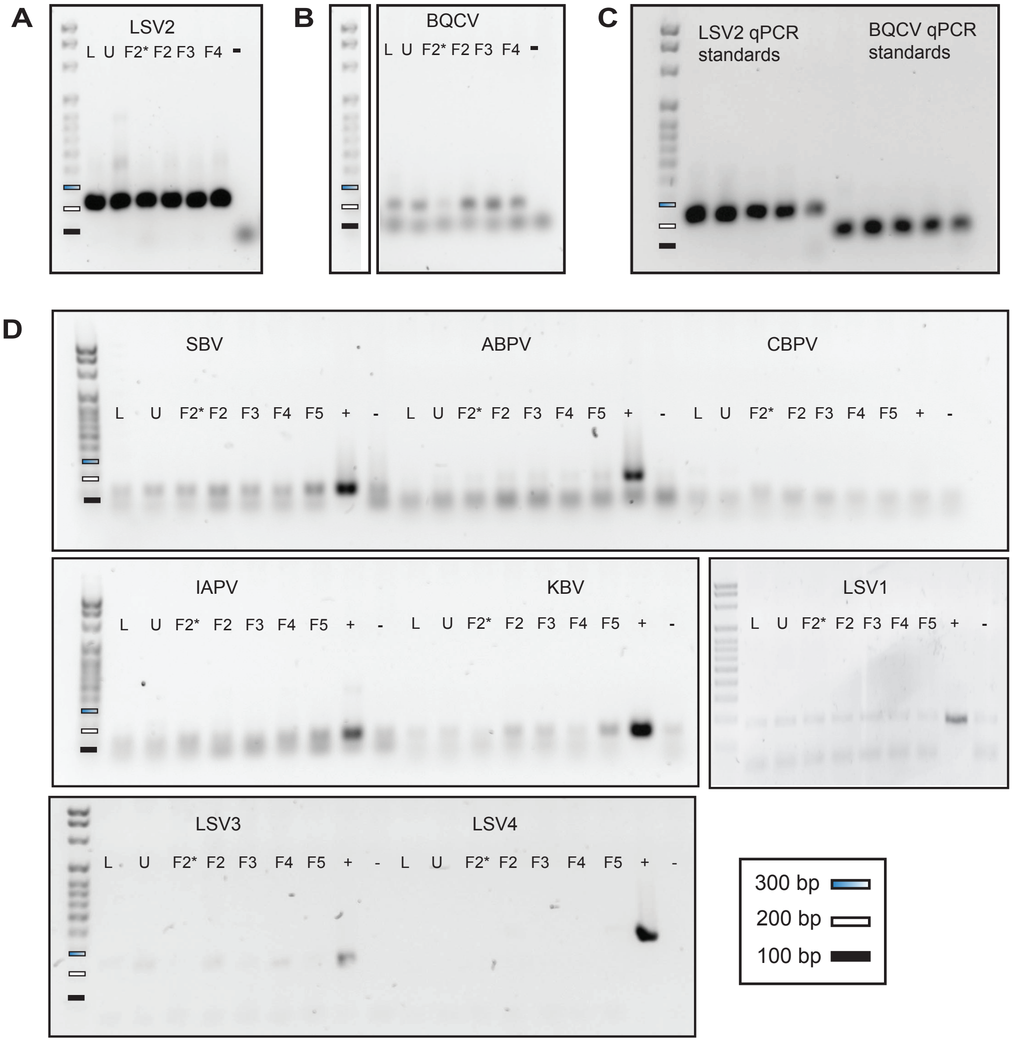

Supplemental Figure S7. Virus Testing of Samples from Lake Sinai virus 2 Purification
Honey bee associated viruses were isolated from a honey bee colony that was primarily infected with LSV2.
The virus purification protocol results in several samples, including the intial honey bee lysate (L),
virus-pellet after ultracentrifugation (U), and several fractions from a CsCl gradient (Fraction 2 unconcentrated (F2*),
concentrated fraction 2 (F2), concentrated fraction 3 (F3), concentrated fraction 4 (F4), and concentrated fraction 5 (F5).
Virus specific PCR was utilized to screen these samples for the presence of common honey bee viruses including:
LSV2, BQCV, SBV, ABPV, CBPV, IAPV, KBV, LSV1, LSV3, and LSV4.
LSV2 and BQCV abundance was assessed by qPCR (see Figure 4), the products were analyzed by agarose gel electrophoresis
(A) LSV2, (B) BQCV, and (C) LSV2 and BQCV qPCR standards 10^9 - 10^5.
(E) Fraction 4 was not positive for SBV, ABPV, CBPV, IAPV, KBV, LSV1, LSV3, or LSV4, agarose gel electrophoresis of
PCR products. Positive and negative control reactions are noted with (+) and (-), respectively.
