# Supplementary material for: Honey Bee Infecting Lake Sinai Viruses
Source: Viruses. 2015 Jun 23;7(6):3285–309. doi: 10.3390/v7062772 (PMC4488739; doi:10.3390/v7062772)
Supplement: Supplementary file 1 [file viruses-07-02772-s001.zip › viruses-07-02772-supplementary/FigS8 qPCR.pdf]

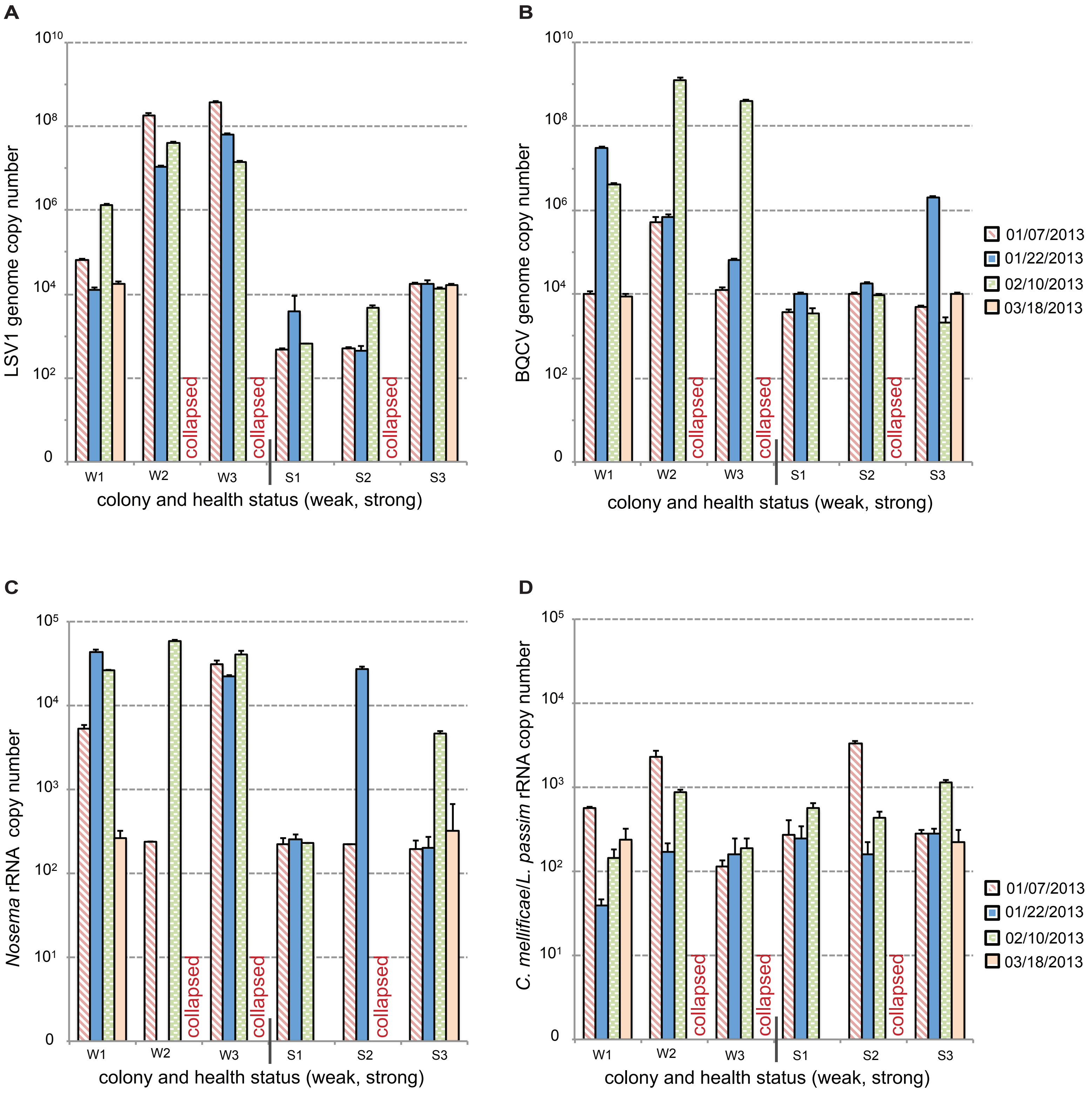

Supplemental Figure S8. LSV1, BQCV, *Nosema*, *C. mellificae* / *L. passim* qPCR data plotted against colony health. Honey bee colony heath and pathogen prevalence and abundance (n=6) was monitored from January – March 2013. Honey bee colonies that were weak (< 5 frames, n=3) at the onset of the study are labeled W1, W2, and W3, and colonies that were healthy (>9 frames, n=3) at the onset of the study are labeled S1, S2, and S3. Quantitative-PCR was used to determine pathogen abundance of **A.** LSV1, **B.** BQCV, **C.** *Nosema ceranae*, and **D.** *C. mellificae* / *L. passim* throughout the course of the study. Overall weak colonies had higher levels of LSV2, LSV1, and BQCV.
