# Supplementary material for: Honey Bee Infecting Lake Sinai Viruses
Source: Viruses. 2015 Jun 23;7(6):3285–309. doi: 10.3390/v7062772 (PMC4488739; doi:10.3390/v7062772)
Supplement: Supplementary file 1 [file viruses-07-02772-s001.zip › viruses-07-02772-supplementary/FigS9 qPCR Stats.pdf]

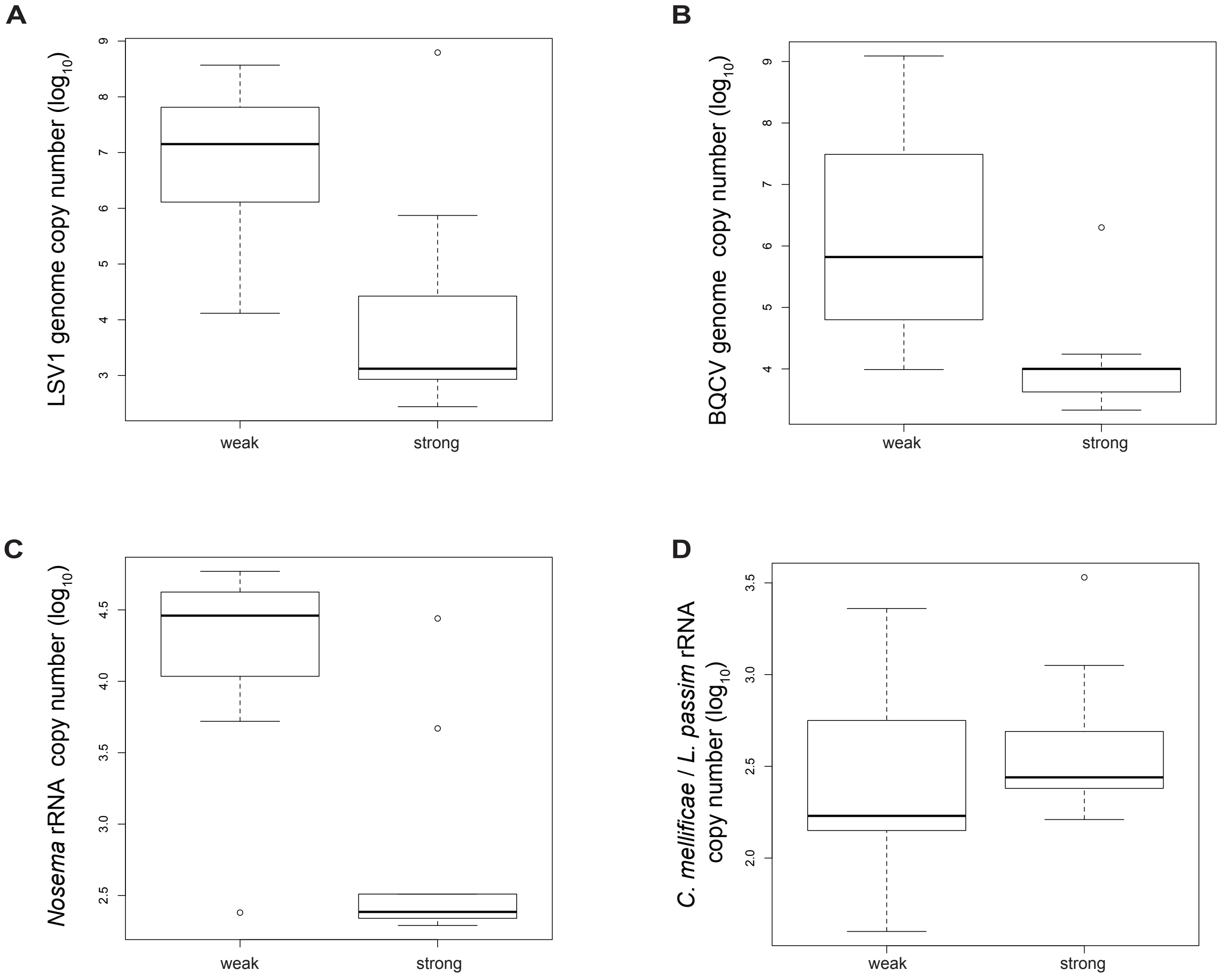

Supplemental Figure S9. Statistical evaluation of LSV1, BQCV, *Nosema*, *C. mellificae* / *L. passim* abundance and colony health. Honey bee colony health, pathogen prevalence (PCR) and pathogen abundance (qPCR) was monitored from January - March 2013. (Supplemental Table S3). Samples were obtained from six honey bee colonies at 3 or 4 different time points (n=6 colonies, 20 total sampling events). Overall weak colonies (< 5 frames, n=9 sampling events) had greater levels of pathogens relative to strong colonies (>9 frames, n=11 sampling events). The mean abundance of LSV2 was greater in weak colonies (log_{10}=7.55) as compared to strong colonies (log_{10}=3.19, p=8.89x10^{-5}) (Figure 7). Likewise weak colonies had a higher mean abundances of (A) LSV1 (weak log_{10}=6.83 vs. strong log_{10}=4.00, p=1.60x10^{-3}), (B) BQCV (weak log_{10}=6.25 vs.strong log_{10}=4.06, p=7.57x10^{-3}), and (C) Nosema ceranae, (weak log_{10}=4.17 vs. strong log_{10}=2.71, p=1.15x10^{-3}), whereas levels of (D) trypanosmatids (*C. mellificae* / *L. passim*) were not statistically different (weak log_{10}=2.40 vs. strong log_{10}=2.60, p=0.35) using a Welch Two Sample t-test to compare the mean pathogen abundance (log_{10} qPCR copy number) in weak versus strong colonies.
