# Supplementary material for: Honey Bee Infecting Lake Sinai Viruses
Source: Viruses. 2015 Jun 23;7(6):3285–309. doi: 10.3390/v7062772 (PMC4488739; doi:10.3390/v7062772)
Supplement: Supplementary file 1 [file viruses-07-02772-s001.zip › viruses-07-02772-supplementary/Supp Table S6.pdf]

	# bees	LSV1	LSV2	LSV3	BQCV	DWV	*C.m./L.p.*	*Nosema*
**Rep 1**	50	1	1	1	1	1	1	1
	25	1	1	1	1	1	1	1
	15	1	1	1	1	1	1	1
	10	1	0	1	1	1	1	1
	5 (set 1)	1	1	1	1	1	1	1
	5 (set 2)	1	1	1	1	1	1	1
	1 (set 1)	1	0	1	1	1	1	1
	1 (set 2)	1	1	1	1	1	1	1

	# bees	LSV1	LSV2	LSV3	DWV	*Nosema*
**Rep 2**	50	1	1	1	0	0
	25	1	1	1	0	1
	15	1	1	1	0	1
	10	1	1	1	0	1
	5 (set 1)	1	1	1	0	0
	5 (set 2)	1	1	1	0	0
	1 (set 1)	1	1	0	0	0
	1 (set 2)	1	1	1	0	0

	# bees	LSV1	LSV2	DWV	*Nosema*
**Rep 3**	30	NA	1	1	0
	5 (set 1)	1	1	1	1
	5 (set 2)	1	1	1	1
	5 (set 3)	1	1	1	0

Supplemental Table S6. Three replicates of pathogen detection in variable numbers of honey bees from a single colony. The quantity of bees indicated in this table were processed as described in Supplemental Figure S10 / Methods. cDNA was prepared from these RNA samples, and was used in PCR for the pathogens listed above. 1 = pathogen detected; 0 = pathogen not detected; NA = pathogen not tested. Data from replicate 1 were presented in Supplemental Figure S10.
