# Supplementary material for: Honey Bee Infecting Lake Sinai Viruses
Source: Viruses. 2015 Jun 23;7(6):3285–309. doi: 10.3390/v7062772 (PMC4488739; doi:10.3390/v7062772)
Supplement: Supplementary file 1 [file viruses-07-02772-s001.zip › viruses-07-02772-supplementary/SuppTable S1 primers.docx]

**_­­_Supplemental Table S1. Primers used in this study.**

| **Genome / Gene Name** | **NCBI #**  **GI #** | **Primer Name** | **Sequence (5’-3’)** | **Product Size (bp)** | **Reference** |
| --- | --- | --- | --- | --- | --- |
| **Ribosomal protein L8 (*Apis m.*)** | XM_393671.5 GI:571556074 | Rpl8Fw  Rpl8Rev | TGGATGTTCAACAGGGTTCATA  CTGGTGGTGGACGTATTGATAA | 121 | Evans et al. (2006) Insect Mol Bio |
| **Lake Sinai virus 1 (LSV1)** | HQ871931  GI:335057596 | qLSV1-F-2569  qLSV1-R-2743** | AGAGGTTGCACGGCAGCATG  GGGACGCAGCACGATGCTCA | 174 | Runckel, Flenniken (2011) PLoS One |
| **Lake Sinai virus 2 (LSV2)** | HQ888865  GI:335057589 | qLSV2-F-1722  qLSV2-R-1947** | CGTGCTGAGGCCACGGTTGT  GCGGTGTCGATCTCGCGGAC | 225 | Runckel, Flenniken (2011) PLoS One |
| **Lake Sinai Virus 3 (LSV3)** | \|  \| JQ480620 \| \| --- \| --- \|   GI:386289721 | LSV3-F-2186  LSV3-R-2429 | CGTGAGCACGATGAGTCAGT  TGGAGGTGCTTGTTGCATAA | 243 | this work |
| **Lake Sinai virus 4 (LSV4)** | JX878492  GI:512134519 | LSV4-F-1896  LSV4-R-2278 | CCATCTCCTCATCCACGTTT  GATTCCCAAATCAGGCTCAA | 379 | this work |
| **Lake Sinai virus 5 (LSV5)** | KC880124  GI:537367126 | LSV5-F-2081  LSV5-R-2270 | TCCGATACTCACGACGAACA  GACACGCGTCAATATCATGG | 190 | this work |
| **black queen cell virus (BQCV)** | AF183905  GI:8100530 | qBQCVorf2F_6664  qBQCVorf2R_6805 | TCCTCAAATCTGGAGCGAAC  GTATTCGCTGGCCGTAAAAC | 141 | Runckel, Flenniken (2011) PLoS One |
| **deformed wing virus (DWV)** | AY292384.1 GI:31540603 | DWVfw1165  DWVrev1338 | CTTACTCTGCCGTCGCCCA  CCGTTAGGAACTCATTATCGCG | 173 | Chen et a. (2005) J Invert Path |
| **sacbrood virus (SBV)** | AF092924.1 GI:4416206 | SBV_F2_5120  SBV_R2_5243 | AATGTCACCCACGAGTGTTG  GCGATGCAACCATACAACTG | 123 | this work |
| ***Crithidia mellificae /***  ***Lotmaria passim*** | PRJNA78249 | qCrFw1  qCrRev1 | TCCACTCTGCAAACGATGAC  GGGCCGAATGGAAAAGATAC | 153 | Runckel, Flenniken (2011) PLoS One |
| ***P. larvae*** | PRJNA30269 | PL2-Fw  PL2-Rev | CGGGAGACGCCAGGTTAG  TTCTTCCTTGGCAACAGAGC | 380 | Marinez et al 2010;  Marinez et al 2011 |
| **acute bee paralysis virus (ABPV)** | NC_002548.1 GI:10314009 | qABPV-F-5457  qABPV-R-5634 | GGATGAGAGAAGACCAATTG  CCAATCTTGGGAATAAACATTAGTTC | 177 | Highfield et al. (2009)  Appl Environ Micro;  Runckel, Flenniken (2011) PLoS One |
| **chronic bee paralysis virus (CBPV)** | NC_010711.1 GI:188543025 | CBPV-F_2580  CBPV-R_3034 | AGTTGTCATGGTTAACAGGATACGAG  TCTAATCTTAGCACGAAAGCCGAG | 454 | Ribiere et al. (2002) Apidologie |
| **Israeli acute paralysis virus (IAPV)** | NC_009025.1 GI:126010924 | IAPV_F_7762  IAPV_R_7876 | GCAGCTATTTTTGGCTGGTC  CCAATGTACGCTCATATCG | 114 | this work |
| **Kashmir bee virus (KBV)** | NC_004807.1 GI:30793779 | KBV_F_4470  KBV_R_4581 | TCGACAAGGACATGATCGAG  GAGCCACAAATGGCTTCTTC | 111 | Stoltz et al. (1995)  J Apicult Res |
| ***Nosema spp.*** |  | Nosema pan rRNA F-322  Nosema pan rRNA R-529 | GGCAGTTATGGGAAGTAACA  GGTCGTCACATTTCATCTCT | 207 | Chen et al. (2008)  J Inv Path |
| ***M. plutonius*** | PRJDA73165 | MelissoF  MelissoR | CAGCTAGTCGGTTTGGTTCC  TTGGCTGTAGATAGAATTGACAAT | 796 | Roetschi et al 2007;  Roetschi et al 2008 |
| **Lake Sinai virus 1 (LSV1)** | HQ871931  GI: 335057596 | LSV-1 5' RACE R-273 | GGATACGCACGCCAGCAGCA |  | this work |
| **Lake Sinai virus 1 (LSV1)** | HQ871931  GI: 335057596 | LSV-1 5' RACE nest R-248 | AGCAACGCGGTGTCTGTG |  | this work |
| **Lake Sinai virus 1 (LSV1)** | HQ871931  GI: 335057596 | LSV-1 3' RACE F-5224 | GCTTGGTGGCAAGGTCTCTGACTTC |  | this work |
| **Lake Sinai virus 1 (LSV1)** | HQ871931  GI: 335057596 | LSV-1 3' RACE nest F-5294 | AAGTAACGGAGACGCAATCTCTGG |  | this work |
| **Lake Sinai virus 2 (LSV2)** | HQ888865  GI: 335057589 | LSV-2 5' RACE R-232 | CACGGTGTACGGTAGCGCAAAATAC |  | this work |
| **Lake Sinai virus 2 (LSV2)** | HQ888865  GI: 335057589 | LSV2 5'RACE nest R-155 | GCCTCAGCAGCAGCATACTC |  | this work |
| **Lake Sinai virus 2 (LSV2)** | HQ888865  GI: 335057589 | LSV-2 3' RACE F-5160 | TGCGTGGAACTGGCTCGGAAATA |  | this work |
| **Lake Sinai virus 2 (LSV2)** | HQ888865  GI: 335057589 | LSV-2 3' RACE nest F-5219 | ACGGCGACATCTTCTTTGAC |  | this work |
| **Lake Sinai virus 4 (LSV4)** | JX878492  GI: 512134519 | LSV4-F-2006 | ACACACGGAGGTCCT |  | this work |
| **Lake Sinai virus 4 (LSV4)** | JX878492  GI: 512134519 | LSV4-F-2129 | CACGCAGATTGCCCC |  | this work |
| **Lake Sinai virus 1 (LSV1)** | HQ871931  GI: 335057596 | LSV1-F-1433-TAGS | GGCCGTCATGGTGGCGAATAACAGGTGCAGAGCAATTGGATTCA |  | Runckel, Flenniken (2011) PLoS One |
| **Lake Sinai virus 2 (LSV2)** | HQ888865  GI: 335057589 | LSV2-F-1433-TAGS | GGCCGTCATGGTGGCGAATAATAGGTGTCGGGCCATAGGGTTTG |  | Runckel, Flenniken (2011) PLoS One |
| **LSV 1 & 2** |  | LSV1&2U-R-1744 | CCATATCATAAGTTGGCAAGTG |  | Runckel, Flenniken (2011) PLoS One |
|  |  | TAGS | GGCCGTCATGGTGGCGAATAA |  | Plaskon et al. (2009) PLoS One |
| **LSV 1 & 2** |  | qLSVU-R-2477 | AGAGGGTACCGCGACACCCATG |  | Runckel, Flenniken (2011) PLoS One |
| **Universal**  **LSV1, 2, 3, 4** |  | LSV1-4-F-2157  LSV1-4-R-2309 | CGTGCGGACCTCATTTCTTCATGT  CTGCGAAGCACTAAAGCGTT | 152 bp | this work |
