# Supplementary material for: Honey Bee Infecting Lake Sinai Viruses
Source: Viruses. 2015 Jun 23;7(6):3285–309. doi: 10.3390/v7062772 (PMC4488739; doi:10.3390/v7062772)
Supplement: Supplementary file 1 [file FigS2 LSV2 5'RACE aligned.pdf › viruses-07-02772-supplementary/FigS2 LSV2 5'RACE aligned.pdf]

Supplemental Figure S2. Lake Sinai virus 2 additional 5'end sequence obtained by sequencing 5' RACE (Rapid Amplification of cDNA ends) product aligned with LSV2 (HQ888865) sequences using Geneious R8 nucleotide alignment. The percent nucleotide identity to LSV1 and LSV2 is shown in the matrix below.

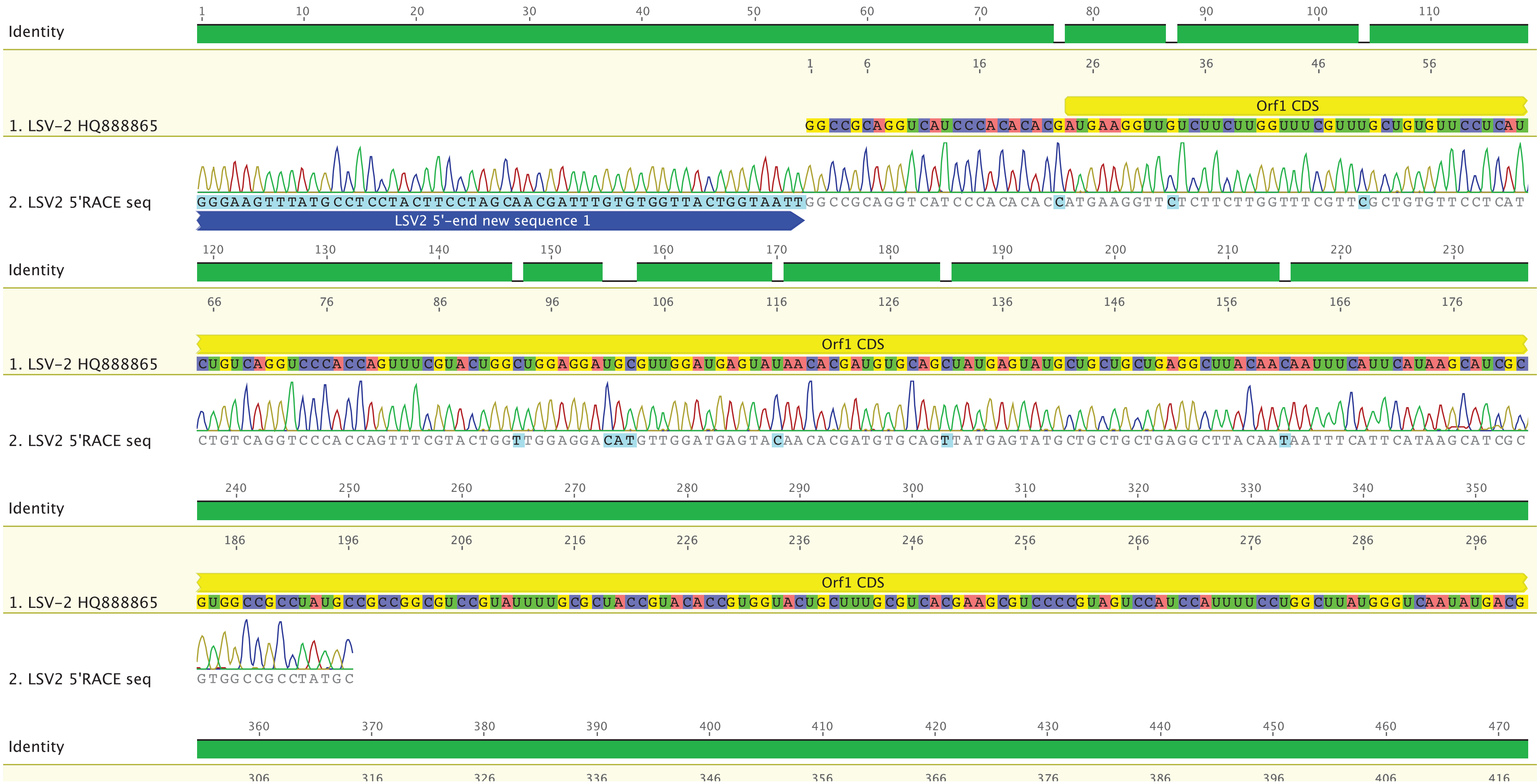

	LSV-2	LSV2 5'RACE	LSV1
LSV-2		94.90	70.29
LSV2 5'RACE	94.90		74.32
LSV1	70.29	74.32	
