# Supplementary material for: Honey Bee Infecting Lake Sinai Viruses
Source: Viruses. 2015 Jun 23;7(6):3285–309. doi: 10.3390/v7062772 (PMC4488739; doi:10.3390/v7062772)
Supplement: Supplementary file 1 [file viruses-07-02772-s001.zip › viruses-07-02772-supplementary/viruses-07-02772-supplementary information.pdf]

Supplementary Information

Supplemental Figure S1. LSV1 and LSV2 negative strand detection

RNA isolated from a LSV containing honey bees lysate was reverse-transcribed with the primer listed below, treated with Exonuclease I to remove excess primer, and amplified using the PCR primers listed for each lane:

(A) LSV1 Data

(L) molecular weight ladder

(1) RT with tagged-negative strand specific LSV1 primer (LSV1-F-1433-TAGS), PCR with TAGS and LSV1 & 2U-R-1744 primers

(2) negative control: no RT in the presence of (LSV1-F-1433-TAGS), PCR with TAGS and LSVSV1 & 2U-R-1744 primers

(3) negative control: RT with tagged-negative strand specific LSV1 primer (LSV1-F-1433-TAGS), PCR with only LSVSV1&2U-R-1744 primer

(4) negative control: RT with random hexamer primer, PCR with TAG and LSVSV1 & 2U-R-1744 primers

(5) negative control: no RT in the presence of random hexamer primer, PCR with LSV1 qPCR primers (qLSV1-F-2569 and qLSV1-R-2743)

(6) positive control: RT with random hexamer primer, PCR with LSV1 qPCR primers (qLSV1-F-2569 and qLSV1-R-2743)

(7) negative control: RT with random hexamer primer, PCR only reverse LSV qPCR primer (qLSV1-R-2743)

(8) evidence of self priming: RT with no primer, PCR with LSV1 qPCR primers (qLSV1-F-2569 and qLSV1-R-2743)

(9) negative control: no template PCR with TAGS and LSV1&2U-R-1744 primers

(10) negative control: no template PCR with LSV1 qPCR primers (qLSV1-F-2569 and qLSV1-R-2743)

(B) LSV2 Data

(L) molecular weight ladder

(1) RT with tagged-negative strand specific LSV2 primer (LSV2-F-1433-TAGS), PCR with TAGS and LSV1 & 2U-R-1744 primers

(2) negative control: no RT in the presence of (LSV2-F-1433-TAGS), PCR with TAGS and LSV1 & 2U-R-1744 primers

(3) negative control: RT with tagged-negative strand specific LSV2 primer (LSV2-F-1433-TAGS), PCR with only LSV1&2U-R-1744 primer

(4) negative control: RT with random hexamer primer, PCR with TAGS and LSV1 & 2U-R-1744 primers

(5) negative control: no RT in the presence of random hexamer primer, PCR with LSV1 qPCR primers (qLSV2-F-1722 and qLSV2-R-1947)

(6) positive control: RT with random hexamer primer, PCR with LSV2 qPCR primers (qLSV2-F-1722 and qLSV2-R-1947)

(7) negative control: RT with random hexamer primer, PCR only reverse LSV2 qPCR primer (qLSV2-R-1947)
(8) evidence of self priming: RT with no primer, PCR with LSV2 qPCR primers (qLSV2-F-1722 and qLSV2-R-1947)
(9) negative control: no template PCR with TAGS and LSV1&2U-R-1744 primers
(10) negative control: no template PCR with LSV2 qPCR primers (qLSV2-F-1722 and qLSV2-R-1947)

Supplemental Figure S2. Lake Sinai virus 2 additional 5′end sequence (GenBank KR022002) obtained by sequencing 5′ RACE (Rapid Amplification of cDNA Ends) product aligned with LSV2 (HQ888865) sequences using Geneious R8 nucleotide alignment [37]. The percent nucleotide identity to LSV1 and LSV2 is shown in the matrix below.

Supplemental Figure S3. Lake Sinai virus 1 additional 3′end sequence obtained by sequencing 3′ RACE (Rapid Amplification of cDNA Ends) products; GenBank KR022003.

Supplemental Figure S4. Lake Sinai virus 2 additional 3′end sequence obtained by sequencing 3′ RACE (Rapid Amplification of cDNA eEnds) products; GenBank KR022004.

Supplemental Figure S5. Lake Sinai virus 4 MT2014 (KR021356), LSV4 (JX878492) and LSV1 (HQ871931) nucleotide sequences were aligned using the Geneious R8 alignment tool [37].

Supplemental Figure S6. Virus RNA dependent RNA polymerase (RdRp) amino acid sequences were aligned generated in Geneious R8 [37] using the MAFFT alignment plugin [53]. GenBank accession numbers are listed in figure and in the methods section.

Supplemental Figure S7. Virus testing of samples from Lake Sinai virus 2 purification

Viruses were isolated from a sample of honey bees that was primarily infected with LSV2. The virus purification protocol results in several samples, including the initial honey bee lysate (L), virus-pellet after ultracentrifugation (U), and several fractions from a CsCl gradient (Fraction 2 unconcentrated (F2*), concentrated fraction 2 (F2), concentrated fraction 3 (F3), concentrated fraction 4 (F4), and concentrated fraction 5 (F5). Virus specific PCR was utilized to screen these samples for the presence of common honey bee viruses including: LSV2, BQCV, SBV, ABPV, CBPV, IAPV, KBV, LSV1, LSV3, and LSV4. LSV2 and BQCV abundance was assessed by qPCR (see Figure 5); the products were analyzed by agarose gel electrophoresis. (A) LSV2; (B) BQCV; and (C) LSV2 and BQCV qPCR standards 10^9–10^5; (D) Fraction 4 was not positive for SBV, ABPV, CBPV, IAPV, KBV, LSV1, LSV3, or LSV4, agarose gel electrophoresis of PCR products. Positive and negative control reactions are noted with (+) and (−), respectively.

Supplemental Figure S8. LSV1, BQCV, *Nosema, C. mellificae*/*L. passim* abundance (qPCR) and colony health

Honey bee colony health and pathogen prevalence and abundance (n = 6) was monitored from January–March 2013. Honey bee colonies that were weak (<5 frames, n = 3) at the onset of the study are labeled W1, W2, and W3, and colonies that were strong (>9 frames, n = 3) at the onset of the study are

labeled S1, S2, and S3. Quantitative-PCR was used to determine pathogen abundance of (A) LSV1; (B) BQCV; (C) *Nosema ceranae*; and (D) *C. mellificae*/*L. passim* throughout the course of the study. Overall weak colonies had higher levels of LSV2, LSV1, and BQCV.

Supplemental Figure S9. Statistical evaluation of LSV1, BQCV, *Nosema, C. mellificae*/*L. passim* abundance (qPCR) and colony health

Honey bee colony health, pathogen prevalence (PCR) and pathogen abundance (qPCR) was monitored from January–March 2013 (Supplemental Table S5). Samples were obtained from six honey bee colonies at three or four different time points (n = 6 colonies, 20 total sampling events). Overall, weak colonies (<5 frames, n = 9 sampling events) had greater levels of pathogens relative to strong colonies (>9 frames, n = 11 sampling events). The mean abundance of LSV2 was greater in weak colonies ($\log_{10}$ = 7.55) as compared to strong colonies ($\log_{10}$ = 3.19, p = 8.89×10^{-5}) (Figure 7). Likewise weak colonies had a higher mean abundances of (A) LSV1 (weak $\log_{10}$ = 6.83 *vs*. strong $\log_{10}$ = 4.00, p = 1.60×10^{-3}); (B) BQCV (weak $\log_{10}$ = 6.25 *vs*.strong $\log_{10}$ = 4.06, p = 7.57×10^{-3}); and (C) *Nosema ceranae*, (weak $\log_{10}$ = 4.17 *vs*. strong $\log_{10}$ = 2.71, p = 1.15×10^{-3}); whereas levels of (D) trypanosmatids (*C. mellificae*/*L. passim*) were not statistically different (weak $\log_{10}$ = 2.40 *vs*. strong $\log_{10}$ = 2.60, p = 0.35) using a Welch Two Sample *t*-test to compare the mean pathogen abundance ($\log_{10}$ qPCR copy number) in weak *versus* strong colonies.

Supplemental Figure S10. Pathogen detection in RNA Samples prepared from variable numbers of honey bees obtained from a single colony

Different quantities of honey bees (50, 25, 15, 10, 5, or 1) were homogenized (*i.e.*, samples of >10 frozen bees were homogenized in sealed plastic bags using a marble rolling pin, then thoroughly mixed by shaking contents, prior to transferring 1 gram of material per sample to a microfuge tube (2 mL) for RNA extraction; samples of <5 bees were homogenized in microfuge tubes (2 mL) using 3mm glass beads (see Methods section for additional details). Following RNA extraction and reverse transcription of a normalized amount of RNA (2,000 ng) from each sample, pathogen-specific PCR was performed to identify the pathogens present in each sample. Samples from this representative colony tested positive for BQCV, DWV, LSV1, LSV2, and *Nosema ceranae*.

A. Images of LSV1, LSV2, DWV, *Nosema ceranae*, and BQCV PCR products after agarose gel electrophoresis; sample sizes (*i.e.*, 50, 25, 15, 10, $5_{(set1)}$, $5_{(set2)}$, $1_{(set1)}$, and $1_{(set2)}$) are noted in each lane.
B. Graphical representation of pathogens detected in each sample. Colored regions of each pie-chart indicate that the pathogen was detected, whereas white areas indicate that the pathogen was not detected in that sample. The results from this representative experiment of three replicates (see Supplemental Table S6), indicate that a sample size of five adequately represents the majority of pathogens associated with a particular colony.

Supplemental Table S1. Primers used in this study

Supplemental Table S2. Nucleotide alignment of all Lake Sinai virus sequences in NCBI (March 2015). Lake Sinai virus nucleotide sequences were aligned using the Geneious Alignment tool with the default cost matrix (65% similarity (5.0 ± 4.0) [49]).

Supplemental Table S3. Amino caid alignment of relevant Lake Sinai virus RdRp sequences

Lake Sinai virus RdRp amino acid sequences were aligned using the Geneious alignment tool. Note that 100% identity in shaded cells is a result of very small regions of overlapping sequences between select LSVs and therefore is not indicative of 100% identity over the entire sequence lengths.

Supplemental Table S4. LSV2 capsid protein validation by mass spectrometry

The putative LSV2 capsid protein band (53.7 kDA, Figure 5C) was excised from the acrylamide gel, subjected to in-gel digestion, and the peptide products were detected via nano-HPLC-ESI mass spectrometry. Protein identification was conducted against a concatenated target/decoy version of a database that included all *Apis* proteins in TrEMBL, all Human proteins in SwissProt, and additional proteins including: Lake Sinai virus 1 (GenBank: AEH26192.1, LSV1 capsid protein GI:335057599, LSV1 orf1 GI:335057597, LSV1 RdRp GI:335057598), Lake Sinai virus 2 (GenBank: HQ888865.1, **LSV2 capsid protein GI:335057591**, LSV2 orf1 GI:335057590, LSV2 RdRp GI:335057592), as well as from the open reading frames of two other honey bee viruses Black queen cell virus (BQCV–2 large poly proteins, NC_003784.1, non-structural protein GI:20451022, structural polyprotein GI:20451023), and DWV (one large poly protein, NC_004830.2, GI:71480056), and one abundant honey bee encoded protein vitellogenin (NM_001011578.1; GI:58585104) complement of the UniProtKB. There were 47,247 target sequences in the queried database and reversing the target sequences created additional decoy sequences. Peptides and proteins were inferred from the spectrum identification results using PeptideShaker (version 0.37.5). Peptide Spectrum Matches (PSMs), peptides and proteins were validated at a 1.0% False Discovery Rate (FDR) estimated using the decoy hit distribution. Excluding known contaminants (*i.e.*, human keratin), a total of eight unique peptides identified LSV2 capsid protein in the staining band (Figure 5C–E). The eight LSV2 corresponding peptides had a Peptide Shaker Score and Confidence of 100, and covered 18.85% of the LSV2 capsid protein sequence (GI: 335057591) (Figure 5E). In addition, one peptide corresponding to *Apis mellifera* Major royal jelly protein 2 (UnitProt/Swiss-Prot 077061) was detected. This protein has a molecular weight similar to the LSV2 capsid protein and is very abundant in bees; therefore it is likely a contaminating protein in our honey bee virus preparation. No significant non-contaminant hits were identified in the control region of the gel.

Supplemental Table S5. Complete PCR and qPCR results from a small colony monitoring project

Honey bee colony health, pathogen prevalence (PCR) and abundance (qPCR) was monitored from January–March 2013. Samples were obtained from six honey bee colonies at three or four different time points (n = 6 colonies, 20 total sampling events). Honey bee colonies that were weak (<5 frames, n = 3) at the onset of the study are labeled W1, W2, and W3, and colonies that were strong (>9 frames, n = 3) at the onset of the study are labeled S1, S2, and S3; colony strengths (*i.e.*, weak (W) or strong (S)) were recorded at each sampling event. List of pathogen specific PCR tests and abbreviations used in this table is as follows: Lake Sinai virus 2 (LSV2), LSV1, Black queen cell virus (BQCV), *Nosema ceranae* (Nos.), *Crithidia mellificae*/*Lotmaria passim* (Tryp.), and Deformed wing virus (DWV). PCR positive results are indicated by '1', negative results are indicated by '0', and not tested with NA. Quantitative PCR was utilized to assess LSV2, LSV1, BQCV, *Nosema ceranae,* and trypanosomatid (*Crithidia mellificae*/*Lotmaria passim*) abundance since the majority of the samples tested positive for these pathogens.
